# The object-color solid

**DOI:** 10.1167/jov.25.2.2

**Published:** 2025-02-03

**Authors:** Alexander D. Logvinenko, Brian Funt, Pouya Bastani

**Affiliations:** 1 Glasgow Caledonian University, Glasgow, UK; 2 School of Computing Science, Simon Fraser University, Vancouver, British Columbia, Canada

**Keywords:** object-color solid, optimal reflectances, set of all colors

## Abstract

An algorithm is described that for the first time accurately computes the true object-color solid. Previous methods have computed only approximations to the true object-color solid since they have been based on Schrödinger’s (partially incorrect) assumption that optimal reflectances contain only two transitions. There are, however, three- and four-transition optimal reflectances and these additional reflectances lead to a larger object-color solid than one based on two-transition reflectances alone. The differences between the approximate and true object-color solids have now been quantified. It is further shown that—despite there being optimal reflectances with up to four transitions—the object-color solid can, nonetheless, be parametrized in terms of only two variables. Finally, a method for solving a previously unsolved problem that Schrödinger posed a century ago is presented. Namely, for any given direction in color space, the algorithm determines the corresponding optimal reflectance.

## Introduction

Light entering the eye invokes a triplet of the cone excitations, and the set of cone excitation triplets arising in response to all possible lights forms a cone (referred to as the *color cone*) in the cone excitation space (Wyszecki & Stiles, 1982; Logvinenko, 2015). A reflecting object is only visible due to the light reflected from it. Under a single illuminant, the set of cone response triplets occurring in response to the lights reflected by all possible objects forms a volume inscribed within the color cone. This volume is commonly referred to as the *object-color solid* (Wyszecki & Stiles, 1982).

Although pictures of the object-color solid can be found in many textbooks (Wyszecki & Stiles, 1982; Maximov, 1984; Koenderink, 2010) they do not represent the object-color solid entirely correctly. As a surface in the three-dimensional space, plotting the object-color-solid boundary requires finding, for each direction (a ray from the origin) in the color cone, where it intersects the object-color-solid boundary. To do this, one needs to determine the spectral reflectance function that maps to this boundary point. Such a reflectance is usually referred to as an optimal reflectance. Solving for the optimal reflectance corresponding to a given direction is a long-standing and unsolved problem for which a solution is described in this article.

It should be emphasized that we propose an algorithm for finding an exact solution (i.e., a solution with an arbitrary predetermined accuracy) not simply an approximation. More specifically, we show how to calculate the spectral reflectance function mapping to an arbitrary boundary point. Such a solution is fundamentally unattainable if instead of true functions (e.g., spectral reflectance functions, cone sensitivity functions) sampling vectors are used because in that case, the solution will also be a sampling vector, not a function.

Of course, sampling vectors are typically used in colorimetry. However, it is not obvious in what sense a finite-dimensional vector can be interpreted as a reflectance, which is actually an infinite-dimensional vector (i.e., a function). Interpreted as a sampled reflectance, the natural question arises as to what values the reflectance function takes between the samples.

By computing the scalar product of each sampled cone sensitivity function with a large number of sampled spectral reflection functions, one can get a volume in the color cone, but it will differ to some extent from the true object-color solid. An important question is whether it can be considered to be an approximation of the true object-color solid. It will be a true approximation if, with an unlimited increase in the number of samples, this approximating volume tends to the true object-color solid in the limit. However, the shape of the true object-color solid has remained unknown. Hence, there is a need to establish its true shape as is done elsewhere in this article.

It is regrettable that the importance of the problem of determining optimal spectral reflectance functions has been underestimated for so long; without solving it, it is impossible to establish the true object-color solid. This is a result of the unfortunate tradition of taking on faith Schrödinger’s mistaken claim that the optimal spectral reflectance functions comprise only the two-transition step functions. However, not every two-transition step function serves as an optimal reflectance function, and there are step functions with more than two transitions that are optimal reflectance functions (Logvinenko & Levin, 2023). As a consequence, plotting a map of all the two-transition step functions in the cone excitation space, as many authors have done (e.g., Wyszecki & Stiles, 1982; Maximov, 1984; Koenderink, 2010) does not result in the true object-color solid but, instead, a somewhat smaller volume inside the true object-color solid. This smaller volume will be referred to as a two-transition approximation of the true object-color solid.

In this article, using some theoretical results formulated earlier (Logvinenko & Levin, 2023), we first describe a method for computing the true object-color solid and then evaluate the difference between it and its two-transition approximation. Although it is reassuring this difference in the cone-excitation space turns out to be rather small, it does not justify ignoring the issue because it is impossible to know how small the difference is going to be until we know what the theoretical object-color solid truly is. Hence, the answer is of interest both from a theoretical perspective and a practical perspective.

## The geometry of the object-color solid

In what follows, we assume that there are three classes of sensors (e.g., the cone photoreceptors or camera sensors) and that the response of the *i*^*th*^ sensor class to a surface of spectral reflectance *x*(λ) illuminated by a light of spectral power distribution *I*(λ) can be expressed as

(1) $$\varphi_{i}\left(x\right)=\int_{\lambda_{\min}}^{\lambda_{\max}}x\left(\lambda \right)I\left(\lambda \right)s_{i}\left(\lambda \right)d\lambda ,$$

where *s*_*i*_(λ) is the spectral sensitivity (responsivity) function of the *i*^*th*^ sensor, and [λ_min _, λ_max _] is the visible spectrum interval. In all our calculations we use λ_min _ = 380 nm and λ_max _ = 780 nm. The triplet (φ_1_(*x*), φ_2_(*x*), φ_3_(*x*)) will be referred to as the *color signal*. The three-dimensional space endowed with Cartesian coordinates (φ_1_, φ_2_, φ_3_) will be referred to as the *color signal space*. As far as human vision is concerned, it is synonymous with the cone excitation space (Smith & Pokorny, 1996). The object-color solid is the set of color signals obtained from all possible spectral reflectance functions *x*(λ) under a given illuminant *I*(λ) (Schrodinger, 1920; Luther, 1927; Nyberg, 1928; Wyszecki & Stiles, 1982; Maximov, 1984; Koenderink & van Doorn, 2003; Logvinenko & Levin, 2023).

From the mathematical point of view, the object-color solid is the image, $\Phi \left(X\right)$, of the map $\Phi :X\to R^{3}$, where Φ = (φ_1_, φ_2_, φ_3_), X is the set of all the spectral reflectance functions (i.e., 0 ⩽ *x*(λ) ⩽ 1), and **R**^3^ is the arithmetic three-dimensional vector space.

The set $\Phi \left(X\right)$ is a convex body (i.e., a closed convex set with nonempty interior and without “holes” inside) (see (Logvinenko & Levin, 2023) for proof). Therefore, the object-color solid $\Phi \left(X\right)$ is fully specified by its boundary surface, denoted $\partial \Phi \left(X\right)$.

The spectral reflectance functions that map to the object-color-solid boundary are called *optimal* (sometimes referred to as *optimal stimuli*) (Wyszecki & Stiles, 1982). The set of all optimal reflectances, that is, the set of spectral reflectance functions *x*_*opt*_, such that $\Phi \left(x_{opt}\right)\in \partial \Phi \left(X\right)$, (written as $X_{opt}$), completely specifies $\partial \Phi \left(X\right)$ and hence $\Phi \left(X\right)$. Therefore, the problem of specifying the object-color solid reduces to the problem of specifying $X_{opt}$.

Logvinenko and Levin (2023) put forth a general approach, which we adopt here, for determining the optimal reflectances for human vision. Briefly, this approach boils down to the following. Let *S* denote the object-color solid and ∂*S* its boundary in the color signal space (i.e., $S=\Phi \left(X\right)$, $\partial S=\partial \Phi \left(X\right)$). Denote the coordinates of the color signal space by *z*_1_, *z*_2_, and *z*_3_. As established in convex analysis, a closed convex set *S* in **R**^3^ can be represented as an intersection of closed half-spaces containing this set (Rockafellar, 1970). Moreover, in such a representation one can consider only so-called supporting half-spaces.

Specifically, recall that a plane divides **R**^3^ into half-spaces, and a half-space is closed if the dividing plane belongs to it. Define a *supporting* plane *H* of *S* as one such that: i) *S* fully belongs to one of the two closed half-spaces determined by *H* (which is called the supporting half-space); and ii) *S* has at least one point, **z**, on plane *H*. If there is just one plane *H* supporting *S* at point **z** then *H* is said to be *tangent* to *S* at point **z**. The corresponding half-spaces are called tangent half-spaces. It has been proven that a closed convex set *S* in **R**^3^ can be represented by the intersection of the supporting (or even only tangent) half-spaces of *S* (Rockafellar, 1970).

Analytically, a supporting plane can be expressed as a level set of a particular linear functional on **R**^3^. The linear functional ϕ: **R**^3^ → **R** is a *supporting functional* of *S* at the point **z**^0^ if ϕ(**z**) ⩽ ϕ(**z**^0^) for every **z** in *S*. The supporting plane *H* to *S* at **z**^0^ is, then, expressed as *H* = ϕ^−1^(ϕ(**z**^0^)). That is, *H* is the level set of ϕ for the number ϕ(**z**^0^).

Any continuous linear functional ϕ on the color signal space achieves its maximum on *S* at some point **z**^0^ (for *S* is a closed and bounded subset in **R**^3^):

(2) $$\max_{z\in S}\left\{\phi \left(z\right)\right\}=\phi \left(z^{0}\right).$$

Clearly, this point cannot lie inside *S* and must belong to the boundary ∂*S*. In other words, the supporting plane determined by ϕ touches the boundary of *S* at **z**^0^. This suggests how boundary points of *S* can be revealed. We need to look for the maxima of all possible linear functionals on the object-color solid *S*. As is well-known, a linear functional on **R**^3^ is determined by three real numbers. We will assume that at least one of these numbers is not zero. Formally, any *k*_1_, *k*_2_, and *k*_3_ specify a linear functional ϕ, the value of which for a vector **z** = (*z*_1_, *z*_2_, *z*_3_) ∈**R**^3^ is given as

(3) $$\phi \left(z\right)=k_{1}z_{1}+k_{2}z_{2}+k_{3}z_{3}.$$

Consider a linear functional ϕ_**k**_(**z**) determined by some given vector **k** = (*k*_1_, *k*_2_, *k*_3_) and let us find a point $z^{0}=\left(z_{1}^{0},z_{2}^{0},z_{3}^{0}\right)$ on the object-color-solid boundary ∂*S* at which ϕ_**k**_(**z**) is maximal. As each *z*_*i*_ (*i* = 1, 2, 3) is the value of φ_*i*_ for some $x\left(\lambda \right)\in X$, linear functional ϕ_**k**_(**z**) induces a corresponding linear functional on X:

(4) $$\begin{array}{c} \;\phi_{k}\left(x\left(\lambda \right)\right)=k_{1}\varphi_{1}\left(x\left(\lambda \right)\right)+k_{2}\varphi_{2}\left(x\left(\lambda \right)\right)+k_{3}\varphi_{3}\left(x\left(\lambda \right)\right),\; \end{array}$$

which in turn can be rewritten as

(5) $$\phi_{k}\left(x\left(\lambda \right)\right)=\int_{\lambda_{\min}}^{\lambda_{\max}}x\left(\lambda \right)G\left(\lambda \right)d\lambda ,$$

with

(6) $$\begin{array}{c} \;G\left(\lambda \right)=k_{1}I\left(\lambda \right)s_{1}\left(\lambda \right)+k_{2}I\left(\lambda \right)s_{2}\left(\lambda \right)+k_{3}I\left(\lambda \right)s_{3}\left(\lambda \right).\; \end{array}$$

It is clear that every spectral reflectance function *x*(λ) that maps to **z**^0^ (i.e., Φ(*x*(λ)) = **z**^0^) must maximize the value of integral (Equation 5). Therefore, the problem of finding the boundary point **z**^0^ amounts to finding spectral reflectance functions in X that maximize the integral in (Equation 5).

Let *B*_+_ = {λ: *G*(λ) > 0}, *B*_0_ = {λ: *G*(λ) = 0}, and *B*_−_ = {λ: *G*(λ) < 0}. It is clear that to maximize (Equation 5), *x*(λ) must be the maximal value (i.e., 1) for all λ ∈ *B*_+_ , and the minimal value (i.e., 0) for all λ ∈ *B*_−_. Because the values of *x*(λ) for λ ∈ *B*_0_ do not affect the integral, *x*(λ) can take any arbitrary value there. In particular, for any subset *B* = *B*_+_∪*B*′, such that *B*′⊆*B*_0_, the function

$$\chi_{B}\left(\lambda \right)=\left\{\begin{array}{ccc} 1, & \text{if} & \lambda \in B;\\ 0, & \text{if} & \lambda \notin B, \end{array}\right.$$

will be an optimal reflectance mapping to **z**^0^ (i.e., Φ(χ_*B*_) = **z**^0^).

In general, there can be many spectral reflectance functions mapping to a given boundary point **z**^0^. For example, included in *B*_0_ is any subset of wavelengths Λ′⊂[λ_min _, λ_max _] for which the illuminant *I*(λ) = 0. As a consequence, any spectral reflectance function that takes 1 on *B*_+_, arbitrary values on Λ′, and 0 at the remaining wavelengths will be an optimal reflectance. In fact, there will be an infinite number of optimal reflectances corresponding to the same color signal **z**^0^. Moreover, because they produce the same color signal **z**^0^, they all will be metameric, which means there will be metamerism on the object-color-solid boundary. If we consider, however, only everywhere positive illuminants (i.e., *I*(λ) > 0 for all λ in [λ_min _, λ_max _]) the situation becomes more interesting. In this case, function *G*(λ) and the following function:

(7) $$g\left(\lambda \right)=k_{1}s_{1}\left(\lambda \right)+k_{2}s_{2}\left(\lambda \right)+k_{3}s_{3}\left(\lambda \right)$$

lead to the same *B*_+_, *B*_0_, and *B*_−_. Indeed, multiplying *g*(λ) by any everywhere positive function of wavelength cannot change the roots, which means that *B*_0_ (thus *B*_+_ and *B*_−_) will remain the same. Therefore, for any everywhere positive illuminant, the set of optimal spectral reflectance functions will be the same for the given spectral sensitivity functions as it will also be for any pre-receptor filter (e.g., atmosphere) with everywhere positive spectral transmittance. In other words, changing the illuminant does not affect the optimal reflectance set unless this change fully filters out some interval of the spectrum. Hence, for all such illuminants and viewing conditions, the optimal reflectance set $X_{opt}$ is solely determined by the spectral sensitivity functions.

Thus, we now have a general method for generating optimal reflectances. Because each vector **k** generates one (or more) optimal reflectances for some boundary point determined by **k**, it is intuitively clear that running through all possible vectors **k** will, in principle, lead to the whole set of optimal reflectances $X_{opt}$ (The formal proof can be found in Logvinenko & Levin, 2023).

In terms of a geometrical interpretation of the sets *B*_+_, *B*_0_ and *B*_−_, consider the spectral curve in color signal space defined by the given set of spectral sensitivity functions:

$$\begin{array}{ccc} \;\vec{C}\left(\lambda \right) & \;= & \left(\varphi_{1}\left(\delta \left(\mu -\lambda \right)\right),\varphi_{2}\left(\delta \left(\mu -\lambda \right)\right),\varphi_{3}\left(\delta \left(\mu -\lambda \right)\right)\right)\\ & \;= & \left(s_{1}\left(\lambda \right),s_{2}\left(\lambda \right),s_{3}\left(\lambda \right)\right) \end{array}$$

where δ(μ − λ) stands for the Dirac delta function centered at wavelength λ. For the purpose of computing the spectral curve, we resort to the common practice of representing monochromatic lights by Dirac delta functions; however, in the rest of the article, such illuminants are excluded from the theory.

Given three real numbers *k*_1_, *k*_2_, and *k*_3_, the following equation

(8) $$k_{1}z_{1}+k_{2}z_{2}+k_{3}z_{3}=0$$

determines a plane in color signal space through the origin. As (*s*_1_(λ), *s*_2_(λ), *s*_3_(λ)) is a point on the spectral curve corresponding to wavelength λ, the equation

(9) $$g\left(\lambda \right)=k_{1}s_{1}\left(\lambda \right)+k_{2}s_{2}\left(\lambda \right)+k_{3}s_{3}\left(\lambda \right)=0$$

can be considered as the condition that it belong to the plane determined by Equation 8. Hence, all the roots of Equation 9 are exactly the points on the spectral curve that belong to that plane, in other words, the points where the plane intersects the spectral curve. *B*_0_, therefore, consists of exactly the set of roots of Equation 9 as well. The crucial result of the above derivation is: If the spectral sensitivity functions are continuous, as is the case for human vision, then the zero-crossings (by a zero-crossing we mean a root in the vicinity of which the function changes sign) of the function *g*(λ) in Equation 7 determine the optimal reflectance generated by the given **k**. See The geometry of the object-color solid for more details.

Obviously, not every plane through the origin intersects the spectral curve $\vec{C}\left(\lambda \right)$. For example, for everywhere positive spectral sensitivity functions, as is again the case for human vision, and **k > 0** (i.e., a vector **k** with positive components *k*_1_, *k*_2_, and *k*_3_) the function *g*(λ) in Equation 7 lies above the horizontal axis and yields no roots. All the planes determined by **k > 0** bring about the same optimal stimulus; namely, the spectral reflectance function that is identically unity (denoted as *x*(λ) ≡ 1) taking the value 1 for every wavelength in [λ_min _, λ_max _]. It will be referred to as the *perfect reflector* *x*_*W*_. The corresponding point on the object-color-solid boundary, Φ(*x*_*W*_) will be called the *white pole* of the object-color solid. Likewise, the planes determined by **k < 0** (i.e., **k** with negative components *k*_1_, *k*_2_, and *k*_3_) all generate the optimal reflectance taking 0 for every λ ∈ [λ_min _, λ_max _] (*x*(λ) ≡ 0). It will be referred to as the *perfect absorber* *x*_*B*_. Φ(*x*_*B*_) will be called the *black pole*.

In fact, for any **k**, if *x*_*opt*_(λ) is an optimal reflectance corresponding to ϕ_**k**_ then the spectral reflectance function 1 − *x*_*opt*_(λ) will be an optimal stimulus corresponding to ϕ_−**k**_. The optimal stimuli *x*_*opt*_(λ) and 1 − *x*_*opt*_(λ) will be referred to as *complementary*. Complementary stimuli are mapped to two points on the object-color-solid boundary that are symmetrical with respect to the center of symmetry of the object-color solid. The spectral reflectance function identically equal to 0.5 (i.e., *x*(λ) ≡ 0.5) will be called *flat gray* and denoted *x*_0.5_. The color signal produced by flat gray, Φ(*x*_0.5_), is located at the center of symmetry of the object-color solid.

It is useful to describe the entire set of parameters **k** that generate one and the same optimal reflectance *x*_*B*_ (respectively, *x*_*W*_) mapping to the black (respectively, white) pole. Recall that the color signals induced by all possible (including monochromatic) lights form what is usually called the color cone. The color cone is the convex hull of the conical surface through the spectral curve (Logvinenko & Levin, 2023; Corollary 4.1). It is a closed cone in **R**^3^. Consider some **k** and the plane determined by it. If the color cone remains entirely on one side of this plane then the spectral curve does not cross this plane, although it may touch it at some points. In other words, Equation 9 can have roots but not zero-crossings. Therefore, such a **k** generates either the perfect absorber, *K*_*B*_, or the perfect reflector, *K*_*W*_. The condition that the color cone is completely contained in the closed half-space determined by **k** can be expressed as either ϕ_**k**_(**z**) ⩽ 0 or ϕ_**k**_(**z**) ⩾ 0 for any **z** belonging to the color cone. For any **k** such that ϕ_**k**_(**z**) ⩽ 0 function *g*(λ) in Equation 7 will be everywhere nonpositive; hence, *B*_−_∪*B*_0_ will be equal to [λ_min _, λ_max _], and thus the perfect absorber will be the optimal stimulus induced by **k**.

Formally, the set of all **k**s generating the perfect absorber, *K*_*B*_, can be represented as

(10) $$K_{B}=\left\{k\in R^{3}:\phi_{k}\left(z\right)\leq 0,\forall z\in K_{C}\right\},$$

where *K*_*C*_ is the color cone. In convex analysis, *K*_*B*_ is called the *polar cone* to the cone *K*_*C*_ (Rockafellar, 1970).

By symmetry, we obtain a similar expression for the set of all **k**s generating the perfect reflector, *K*_*W*_:

(11) $$K_{W}=\left\{k\in R^{3}:\phi_{k}\left(z\right)\geq 0,\forall z\in K_{C}\right\}.$$

As one can see, *K*_*W*_ = −*K*_*B*_, that is, *K*_*W*_ is equal to the negative of the polar cone for the color cone. The polar cone for the color cone is closed and convex. Its boundary is composed of the functionals supporting the color cone. Geometrically, it is fully characterized by the supporting planes of the color cone.

A supporting plane of the color cone at its vertex is, in fact, a supporting plane of the object-color solid at its black pole as well, and as a result we have a singularity there (Logvinenko & Levin, 2023; Proposition 14.2). Indeed, the vertex of the object-color solid at the origin is rather sharp. By symmetry, the white point is also a singular point of the object-color solid, the singularity being of the same type as for the black pole.

## Optimal reflectances for human vision

In this section, we apply the theory outlined to derive the optimal spectral reflectance functions from the spectral sensitivity functions for the human photoreceptors. Following established practice (Judd & Wyszecki, 1975; Smith & Pokorny, 2003; Logvinenko & Levin, 2023), the cone photoreceptor sensitivity functions *s*_*i*_(λ) will be factored as

(12) $$s_{i}\left(\lambda \right)=t\left(\lambda \right)p_{i}\left(\lambda \right),$$

where *t*(λ) is the transmittance spectrum of the ocular media (e.g., the lens, macular pigment, etc.), and *p*_*i*_(λ) is the spectral absorptance of the *i*^*th*^ photopigment (*i* = 1, 2, 3). As the transmittance spectrum of the ocular media is positive over [λ_min _, λ_max _] (i.e., *t*(λ) > 0 in Equation 12), Equation 9 is equivalent to

(13) $$\;k_{1}p\left(\lambda ;\lambda_{S}^{\max}\right)+k_{2}p\left(\lambda ;\lambda_{M}^{\max}\right)+k_{3}p\left(\lambda ;\lambda_{L}^{\max}\right)=0,$$

where *p*(λ; λ^max ^) is the cone-photopigment absorptance spectrum as defined below in Equation 14, and $\lambda_{S}^{\max}$, $\lambda_{M}^{\max}$, and $\lambda_{L}^{\max}$ are the peak absorbance wavelengths for the short (S), middle (M), and long (L) wavelength sensitive cones, respectively.

**Figure 1.**
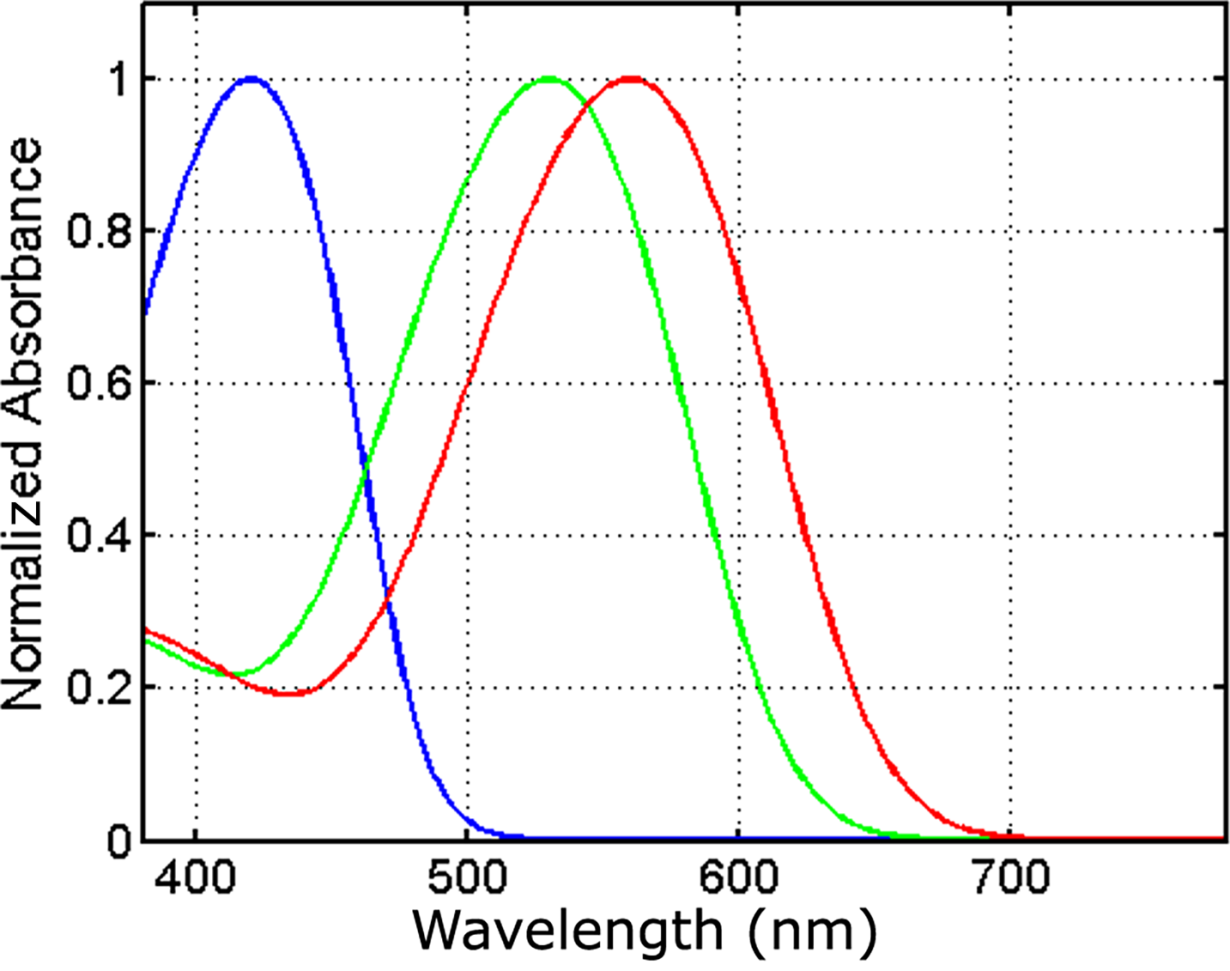
Cone photopigment spectral absorptance curves, scaled to have unit maxima, for the photopigments with peak sensitivity λ^max ^ at the wavelengths of 430, 530, and 560 nm.

The absorptance spectra of the cone photopigments are known and well documented (Dartnall, Bowmaker, & Mollon, 1983; Lamb, 1999; Govardovskii, Fyhrquist, Reuter, Kuzmin, & Donner, 2000). They are commonly described as:

(14) $$p\left(\lambda ;\lambda^{\max}\right)=1-10^{-D_{\max}G\left(\lambda^{\max}/\lambda \right)},$$

where *G*(λ^max ^/λ) is the normalized photopigment absorptivity spectrum, *D*_max _ is the peak photopigment absorbance, and λ^max ^ is the photopigment peak absorbance wavelength. In the present report, we adopt the model of photopigment optical density spectrum put forward by Govardovskii et al. (2000) using *D*_max _ = 0.3, and $\lambda_{S}^{\max}=430$ , $\lambda_{M}^{\max}=530$, and $\lambda_{L}^{\max}$ 560 nm for the S-, M-, and L-cones respectively. These values are in line with the electrophysiological studies of the cones in the macaque and human retina (Schnapf & Schneewels, 1999). The spectral sensitivities of the three cone photopigments calculated using the Govardovskii model with these values are shown in Figure 1. It is worth noting that the resulting spectral sensitivities are not very sensitive to the precise parameters used. The color signal space based on these cone photopigment absorptance spectra will be further referred to as *SML* space, and the coordinates in it as *SML* coordinates.

The function *p*(λ; λ^max ^) in Equation 14 is smooth, and its shape is such that Equation 13 has a finite number of roots. As a consequence, the optimal spectral reflectance functions are elementary step functions. Following the terminology adopted by previous authors (Maximov, 1984; Logvinenko & Levin, 2023), an *elementary step function of degree 1 and type T1* (written as *x*_1_(λ; λ_1_)) is defined as a function of the form

(15) $$x_{1}\left(\lambda ;\lambda_{1}\right)=\left\{\begin{array}{ccc} 0, & \text{if} & \lambda <\lambda_{1},\\ 1, & \text{if} & \lambda \geq \lambda_{1}. \end{array}\right.$$

An *elementary step function of degree 1 and type T2* is defined as

(16) $$1-x_{1}\left(\lambda ;\lambda_{1}\right).$$

Generally, functions of the form

(17) $$x_{m}\left(\lambda ;\lambda_{1},...,\lambda_{m}\right)=\sum_{i=1}^{m}{\left(-1\right)}^{i-1}x_{1}\left(\lambda ;\lambda_{i}\right),$$

and

(18) $$1-x_{m}\left(\lambda ;\lambda_{1},...,\lambda_{m}\right),$$

where λ_min _ < λ_1_ < λ_2_ < … < λ_*m*_ < λ_max _, will be called *elementary step functions of* *degree* *m and type T1 and T2* respectively, with λ_1_,…, λ_*m*_ being referred to as *transition wavelengths*. For the sake of generality, the perfect reflector and the perfect absorber will be referred to as elementary step functions of degree 0 of type T1 and type T2, respectively.

Elementary step functions with the same transition wavelengths, but of different types, are complementary and map to the opposite ends of the interval through the center of the object color solid. It follows from the theory presented in Introduction that if λ_1_ <,…, <λ_*m*_ are the only zero-crossings of the function in Equation 13 (for *k*_1_, *k*_2_, and *k*_3_ at least one of which is not zero) then both the elementary step functions with transition wavelengths λ_1_,…, λ_*m*_ (namely, *x*_*m*_(λ; λ_1_,…, λ_*m*_) and 1 − *x*_*m*_(λ; λ_1_,…, λ_*m*_)) will be the corresponding optimal spectral reflectance functions.

Nearly a century ago, Schrödinger claimed that for human vision the optimal spectral reflectance functions i) do not depend on illuminant, ii) take only values one or zero, and iii) are elementary step functions of degree *m* < 3. However, as we can see now, all three claims are not correct in general. In particular, all the claims fail if *B*_0_ = {λ: *G*(λ) = 0} (where *G* as in Equation 6) is an interval of wavelengths. Still, for a strictly positive illuminant and linearly independent spectral sensitivity functions, the first two claims do hold; although strictly speaking, the second claim is also incorrect because at the wavelengths where a zero-crossing occurs, one can assign any value to the optimal reflectance. However, it seems sensible not to distinguish reflectance functions that differ at only a finite number of wavelengths.

Although the third claim that optimal reflectances do not have more than two wavelength transitions has become a dogma of color science (e.g., MacAdam, 1935; Wyszecki & Stiles, 1982; Koenderink, 2010), it is, nonetheless, not true for the human spectral sensitivity functions, as has been pointed out by some previous authors (West & Brill, 1983; Maximov, 1984; Logvinenko, 2009). Indeed, Equation 13 will have at most two solutions in the visible spectrum interval [λ_min _, λ_max _] only if for every set of distinct λ_1_, λ_2_, λ_3_ in [λ_min _, λ_max _] the following condition holds (Logvinenko and Levin (2023) p. 362):

(19) $$\;\left|\begin{array}{ccc} p\left(\lambda_{1};\lambda_{S}^{\max}\right) & p\left(\lambda_{2};\lambda_{S}^{\max}\right) & p\left(\lambda_{3};\lambda_{S}^{\max}\right)\\ p\left(\lambda_{1};\lambda_{M}^{\max}\right) & p\left(\lambda_{2};\lambda_{M}^{\max}\right) & p\left(\lambda_{3};\lambda_{M}^{\max}\right)\\ p\left(\lambda_{1};\lambda_{L}^{\max}\right) & p\left(\lambda_{2};\lambda_{L}^{\max}\right) & p\left(\lambda_{3};\lambda_{L}^{\max}\right) \end{array}\right|\neq 0\text{,}$$

where |*A*| stands for the determinant of a matrix *A*. In fact, Schrödinger implicitly assumed that this condition (Equation 19) to be satisfied for human vision. If it were to be satisfied then the two-transition assumption would be correct. However, as pointed out by Maximov as early as 1984, and as shown elsewhere in this article, condition (Equation 19) is not always satisfied. In particular, the determinant in Equation 19 for the Govardovskii et al. (2000) absorptance spectra (Equation 14) is 0 for many wavelength triplets.

**Figure 2.**
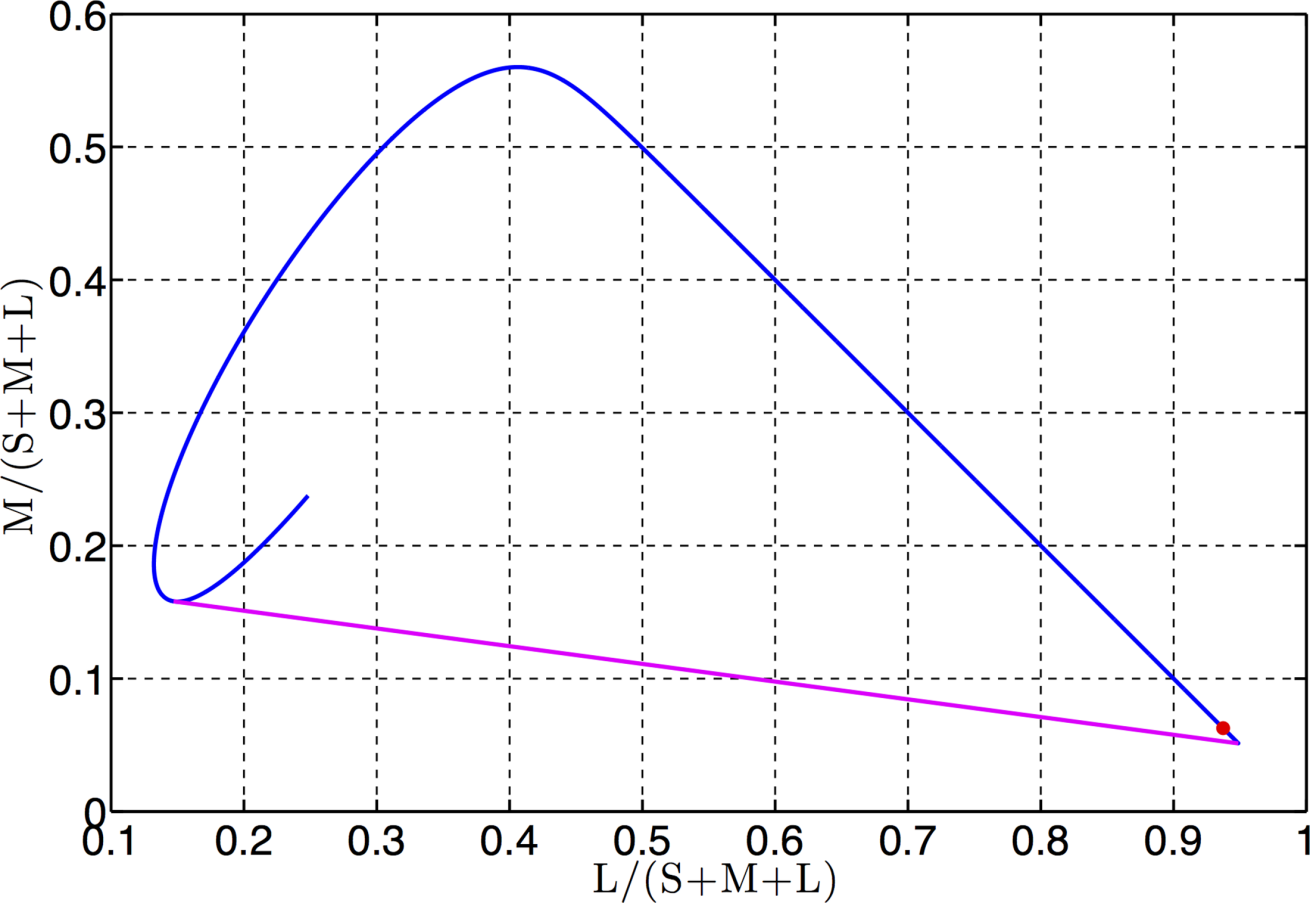
Spectral locus (*blue*) in the chromaticity diagram. Axes are the chromaticity coordinates. S, M, and L are the responses of the short, middle and long-wave sensitive cones (i.e., the *SML* coordinates). The red dot indicates the long wavelength end of the spectral locus. Straight line (*purple*) delimits the boundary of possible chromaticities.

The situation becomes clear when viewed in a chromaticity diagram as shown in Figure 2. Any plane through the origin in color signal space is represented by a straight line in the chromaticity diagram (unless it is parallel to the chromaticity plane). From the diagram it would appear that some such lines in the chromaticity plane could intersect the *spectral locus* (i.e., the projection of the spectral curve on the chromaticity plane) at more than two points. Introducing the chromaticity coordinates *c*_*i*_(λ) = *s*_*i*_(λ)/(*s*_1_(λ) + *s*_2_(λ) + *s*_3_(λ)) as usual, the spectral locus is then defined as the curve $\vec{c}\left(\lambda \right)=\left(c_{1}\left(\lambda \right),c_{2}\left(\lambda \right)\right)$). As a result, it is immediately clear that the two-transition assumption does not hold.

More specifically, some lines intersecting the hook in the short-wavelength (left-hand side in the plot) end of the spectral locus can intersect it more than twice. Furthermore, although not visible in the diagram, there are, in fact, places where a line might intersect the spectral locus at four points. This happens because the spectral locus bends and eventually self-intersects in the long-wavelength region. (See Appendix A for more details.) Let us denote the point of return as $\lambda_{\max}^{'}$. Admittedly, the value of $\lambda_{\max}^{'}$ varies with the peak absorbance wavelengths $\lambda_{S}^{\max}$, $\lambda_{M}^{\max}$, and $\lambda_{L}^{\max}$ (see Equation 14) (Logvinenko, 2015). The value of 700 nm has been obtained for the peak absorbance wavelengths 430, 530, and 560 nm used throughout the article. In other words, the part of the spectral curve corresponding to wavelengths beyond 700 nm almost, but not quite, projects onto the branch of the spectral locus in the chromaticity plane that corresponds to wavelengths under 700 nm. The red dot in Figure 2 marks the end of the visible spectrum λ_max _ = 780 nm, where it, in fact, almost coincides with the point on the spectral locus corresponding to λ = 664 nm. Therefore, the segment of the spectral locus corresponding to the wavelength interval [700, 780] bends back so as to almost overlap with the segment from [664, 700] in Figure 2. This means that there are two hooks in the spectral locus, one at each end of the wavelength spectrum. As a result, a straight line intersecting one of the hooks can also make one more intersection with the spectral locus, thereby indicating that the optimal elementary reflectance step function will have 3 wavelength transitions. A line simultaneously intersecting both hooks will make four intersections with the spectral locus, indicating a four-transition optimal reflectance. Thus, contrary to Schrödinger’s belief, the two-transition assumption is invalid for human vision.

Note that from Equation 9 it follows that a set of cone sensitivity functions will induce the same set of optimal reflectances as any linear (nonsingular) transformation of them. It is also commonly believed that the color matching functions are linearly related to the corresponding cone spectral sensitivity functions (e.g., Stockman, 2019). Although this belief can be challenged from the theoretical point of view (Logvinenko & Levin, 2023), for all practical purposes, color matching functions can be used to estimate optimal reflectances to a good first approximation. Roughly speaking, the optimal reflectances can be expected to remain practically the same irrespective of whether they are derived from the color matching functions, the cone sensitivity functions, or the cone photopigment spectra (the latter two being equivalent because the roots of Equation 13 are the same as those of Equation 9).

However, this is only the case if continuous color matching functions are used, which has never been the case. By their very nature, color matching functions can be only evaluated for some finite number of discrete wavelengths. Furthermore, because color-matching experiments are very time consuming, the number of wavelengths sampled is limited, and has usually not exceeded 35. Even more important is that these wavelengths are predetermined and fixed. That is, the sampling cannot be changed. This alone makes the color matching functions (as well as the spectral sensitivity functions) based on such discrete sampling inappropriate for calculating optimal reflectances. Indeed, the latter reduces to solving Equation 9, which only makes sense for *s*_*i*_ that are continuous functions. Therefore, if, as noted elsewhere in this article, optimal reflectances cannot accurately be determined using only a discrete sample of the values of the spectral sensitivity functions, then it is a fortiori impossible with a discrete sample of the values of the color matching functions as well.

**Figure 3.**
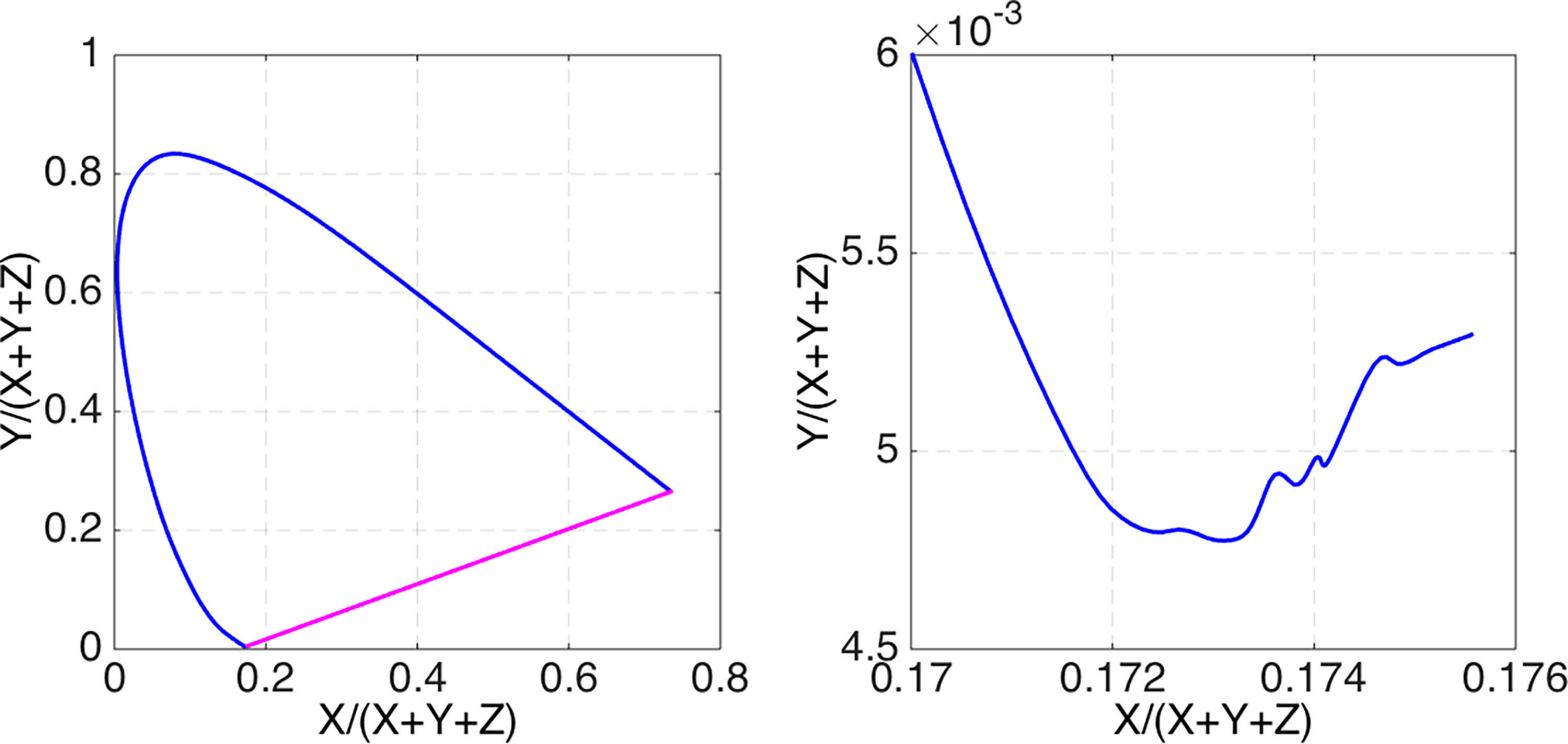
Left: CIE 1931 spectral locus (*blue*). Straight line (*purple*) delimits the boundary of possible chromaticities. Right: zoomed view of the short wavelength segment of the spectral locus.

Worse still, dealing with a coarse sample of the color-matching function values, one can come to incorrect conclusions, even about some qualitative aspects of the set of optimal reflectances such as the number of wavelength transitions. For instance, as West and Brill (1983) previously pointed out, because the number of transitions in the optimal reflectances corresponds to the number of intersections a straight line makes with the spectral locus, the two-transition assumption, therefore, is equivalent to assuming that the chromaticity gamut (i.e., the spectral locus completed with the purple interval) is convex. At first glance, the chromaticity gamut of the CIE 1931 standard observer (Figure 3 left) seems to be convex. Nevertheless, the chromaticity gamut on an extended scale is not convex. At a finer and extended scale, a residual short-wavelength hook (see Figure 3, right) is clearly visible. Although it is not as pronounced as in Figure 2, it is pronounced enough to rule out the convexity of the chromaticity gamut. The reason for the small pronouncedness is that the CIE 1931 color matching functions (thus the chromaticity gamut in Figure 3) are based on data collected only for 400 nm to 700 nm. Furthermore, the step size of 10 nm is too coarse to observe reliably the presence of the hook. Interestingly, the short-wavelength hook in the chromaticity gamut based on more recent (and more accurately measured) color-matching functions, such as those accepted by the CIE as the new standard (e.g., Stockman, 2019), is much more distinct than that in Figure 3, and is more in accordance with the corresponding hook in Figure 2.

One might argue that dealing with a finite-dimensional vector of sample values of color matching functions is usually accompanied by some sort of continuous interpolation (e.g., spline interpolation) of these values. However, such interpolation does not ameliorate the problem that solving Equation 9 presents. Consider Figure 3 (right), where the zigzag-shaped, seemingly continuous, short-wavelength end of the spectral locus arises as a result of smoothing, interpolation and extrapolation of the discrete data. As a consequence, it is easy to see that there are many straight lines in the chromaticity plane intersecting this spectral locus at as many as six (possibly more) points. Needless to say, all these intersections are simply artifacts of the interpolation of the discrete data.

Admittedly, there are more recent color-matching functions which are more elegantly approximated with smooth functions. For example, Stockman and Rider (2023) provide an approximation of the cone spectral sensitivities derived from discrete color-matching data and using Fourier polynomials of the eighth order. As far as the optimal stimuli are concerned, the spectral locus based on these approximations looks consistent with that in Figure 2. However, as the authors themselves emphasize, these approximations are “purely descriptive.” Moreover, they are far from being unique. Given a finite sample of wavelengths (e.g., the 31 CIE 1931 data points), there are infinitely many smooth approximating functions satisfying the data, each leading to a different chromaticity gamut, and thus to different sets of optimal reflectances.

Strictly speaking, there can be no objective criterion for preferring any such set of optimal reflectances over another, which means that formal approximations of discrete data from psychophysical experiments averaged over a sample of subjects (e.g., as done for the CIE 1931 color matching functions) will never suffice. This is why we use the continuous photopigment absorptance curves provided by Govardovskii et al. (2000). The Govardovskii photopigment absorptance functions are based on a physical model of photon capture that provides continuous functions (continuous in principle, not simply continuous extrapolations of discrete samples) describing each cone type.

To perform our calculations, we had to choose a particular set of parameters, specifically the peak absorbance wavelengths. Although these parameters specify the particular absorptance functions of one specific observer, we believe they are fairly representative of human color vision. Generally speaking, in the future, one should take the average (or better, the median) value of these parameters for some sufficiently large sample of human photoreceptors. It is worth noting that averaging the cone photoreceptor sensitivity functions (not to mention the color-matching functions) will not do, because the averaged cone photoreceptor sensitivity functions, *s*(λ), will no longer be represented as a product of the averaged transmittance spectrum of the ocular media, *t*(λ), and the averaged spectral absorptance of the photopigment, *p*(λ), as in Equation 12. As a result, averaging will result in a contaminated set of optimal reflectances, that is, a set that no real human observer will actually have.

## Parameterization of the optimal reflectances

Because the optimal reflectances map to the object-color-solid boundary, which is itself homeomorphic to (i.e., can be bijectively and continuously transformed into) the surface of a sphere, it is clear that one can represent the optimal reflectances for trichromatic vision using only two parameters, even though their degree will generally exceed two. In particular, the object-color-solid boundary can be described in spherical coordinates, which can be used to provide a two-parameter representation, not only for the object-color-solid boundary, but also for the optimal reflectances. The optimal reflectances mapping to the same color signal can differ at no more than four wavelengths. To ignore any such difference, one can agree always to assign 0 reflectance to these wavelengths (i.e., to *B*_0_). This will mean there will be a one-to-one correspondence between the optimal stimuli and the points on the object-color-solid boundary.

Here we consider two approaches to two-parameterization of the optimal reflectances for the trichromatic vision: the first is a spherical representation in the space of *k*-coefficients involved in Equation 6; the second is in terms of transition wavelengths.

### **k**-Parameterization

Consider the arithmetic linear space **R**^3^ with coordinates *k*_1_, *k*_2_ and *k*_3_ (to be referred to as **k**-space). Interpreting these coordinates as coefficients in Equation 6, each **k** = (*k*_1_, *k*_2_, *k*_3_) determines an optimal spectral reflectance function. Evidently, one can consider only unit vectors **k**,

(20) $${∥k∥}_{2}=1.$$

Equation 20 defines a unit sphere in **k**-space. Thus, we have a map of the sphere (Equation 20) onto the optimal reflectances. From the discussion about the poles, we already know this map is not bijective (i.e., not one-to-one). The area corresponding to *K*_*B*_ maps to a single point representing the perfect absorber, as does the area corresponding to *K*_*W*_, which maps to a single point representing the perfect reflector. Denote these closed areas *A*_*B*_ and *A*_*W*_, respectively. They are fully specified by their boundary contours, which are defined by the intersections of the polar cone, and its negative, with the unit sphere. These cones can be evaluated as follows.

As stated the tails of the spectral curve induced by the spectral sensitivity functions based on the Govardovskii absorptance spectra bend into the color cone. Therefore, only a portion of the spectral curve, not the entire spectral curve, belongs to the color cone boundary. The wavelength interval corresponding to this portion, denoted $\left[\lambda_{\min}^{'},\lambda_{\max}^{'}\right]$, is referred to as the *effective visible spectrum interval*. The boundary of the color cone is formed by the conical surface through the effective visible spectrum interval along with the straight interval connecting its ends. These ends were found to be $\lambda_{\min}^{'}=420.8$ nm and $\lambda_{\max}^{'}=700.5$ nm. These values of $\lambda_{\min}^{'}$ and $\lambda_{\max}^{'}$ have been obtained for $\lambda_{S}^{\max}=430$ , $\lambda_{M}^{\max}=530$, and $\lambda_{L}^{\max}$ 560 nm. Of course, a different choice of the peak absorbance wavelengths will bring about somewhat different values of the ends of the effective visible spectrum interval (Logvinenko, 2015).

In terms of color signal coordinates *z*_1_, *z*_2_, and *z*_3_, the plane passing through the origin and tangent to the smooth spectral curve at point $\vec{C}\left(\lambda_{0}\right)$ is defined by the following equation:

(21) $$\left|\begin{array}{ccc} z_{1} & z_{2} & z_{3}\\ s_{1}\left(\lambda_{0}\right) & s_{2}\left(\lambda_{0}\right) & s_{3}\left(\lambda_{0}\right)\\ s_{1}^{'}\left(\lambda_{0}\right) & s_{2}^{'}\left(\lambda_{0}\right) & s_{3}^{'}\left(\lambda_{0}\right) \end{array}\right|=0.$$

Here, $s_{i}^{'}\left(\lambda_{0}\right)$ is the derivative of the *i*^*th*^ spectral sensitivity function at λ_0_. This tangent plane can be expressed as in Equation 8 with the coefficients *k*_1_, *k*_2_, and *k*_3_ determined as

(22) $$k_{i}\left(\lambda_{0}\right)={\left(-1\right)}^{i-1}\left|\begin{array}{cc} s_{p}\left(\lambda_{0}\right) & s_{q}\left(\lambda_{0}\right)\\ s_{p}^{'}\left(\lambda_{0}\right) & s_{q}^{'}\left(\lambda_{0}\right) \end{array}\right|,$$

where *p*, *q* = 1, 2, 3 such that *p*, *q* ≠ *i*.

**Figure 4.**
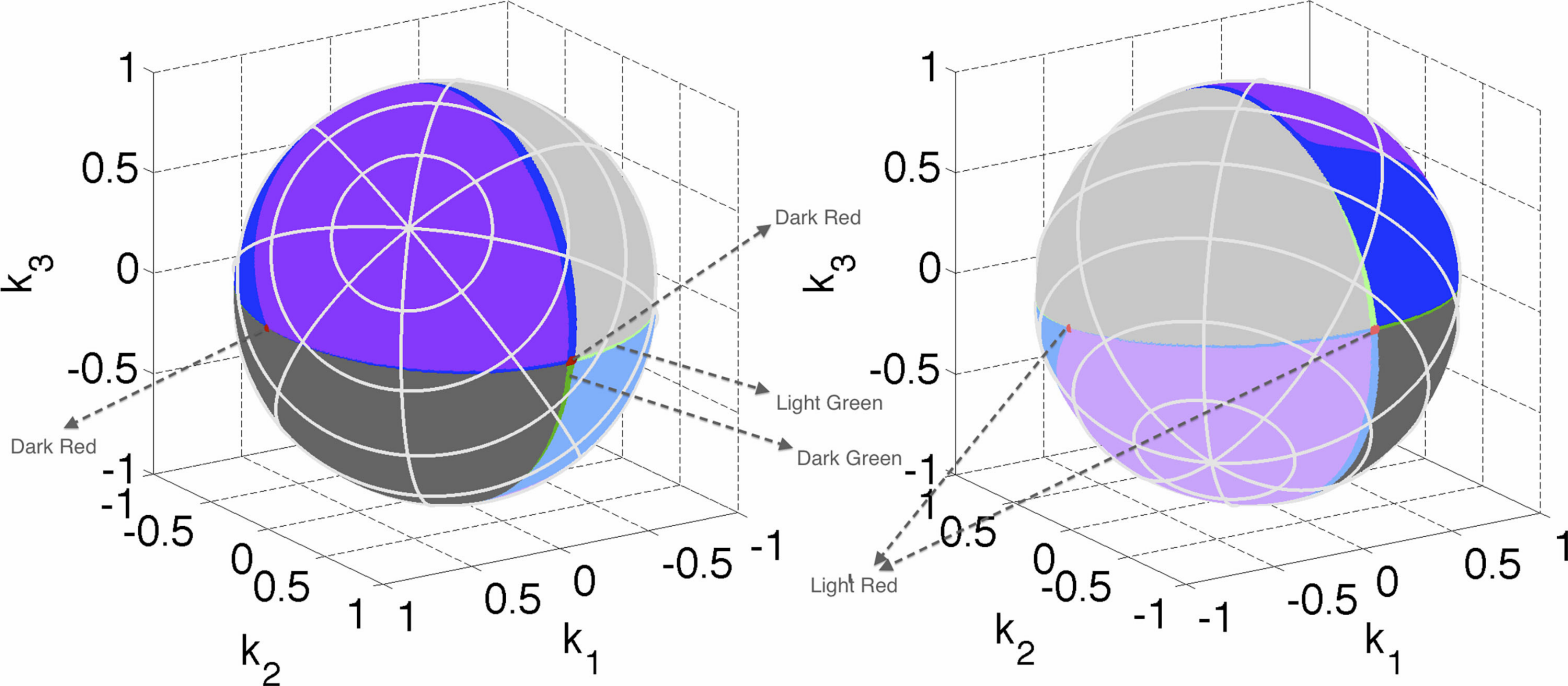
The unit sphere in **k**-space shown in two different orientations with the associated number of transitions of the corresponding optimal reflectance functions plotted in dark/light purple for zero transitions, dark/light gray for one transition, dark/light blue for two transitions, and dark/light green for three transitions, and dark/light red for four transitions. The dark colors correspond to type T1, the light colors to type T2. The poles correspond to **k** = ±[1, 1, 1].

As $k\left(\lambda_{\min}^{'}\right)=k\left(\lambda_{\max}^{'}\right)$ (Logvinenko, 2015), when λ_0_ runs over the effective visible spectrum interval, the set of **k**s determined by Equations 21 and 22 makes a closed curve. A conical surface through this curve in **k**-space is the boundary of the polar cone. The corresponding area, *A*_*B*_, on the unit sphere is colored dark purple in Figure 4. The area *A*_*W*_ is located symmetrically with respect to the center, and is indicated by light purple in Figure 4 (right).

Besides the areas *A*_*B*_ and *A*_*W*_ surrounding the poles, there are two other areas on the sphere where bijectivity fails. These areas correspond to the **k**s that generate one-transition spectral reflectance functions. When stepping through wavelengths from one end of the visible spectrum to the other, while for each wavelength determining **k** along with the roots of the corresponding *g*(λ) from Equation 7, one finds that, for any wavelength λ_0_ between 455.4 nm and 664 nm (again, it is worth remembering that these values have been obtained for $\lambda_{S}^{\max}=430$ , $\lambda_{M}^{\max}=530$, and $\lambda_{L}^{\max}$ 560 nm), there is a plane that intersects the spectral curve only once at $\vec{C}\left(\lambda_{0}\right)$. Any plane crossing the spectral curve at a point outside the interval from 455.4 nm to 664 nm crosses the spectral curve at least twice. It follows that the elementary step functions *x*_1_(λ; λ_1_) of degree 1 such that 455.4 nm <λ_1_ < 664 nm (and the functions complementary to them, 1 − *x*_1_(λ; λ_1_)) exhaust the optimal reflectances having one wavelength transition (for more detail see Appendix A). Note, however, that for wavelength λ_1_ in this interval there is a whole bundle of planes intersecting the spectral curve $\vec{C}\left(\lambda \right)$ only at point $\vec{C}\left(\lambda_{1}\right)$. They all determine the same root in Equation 7, which in turn defines two complementary optimal reflectances with the single transition wavelength λ_1_. In other words, for each λ_1_ ∈ [455.4 ; 664], there is a curved interval on the sphere that maps to *x*_1_(λ; λ_1_). The set of all such curvilinear intervals for all wavelengths in [455.4 ; 664] makes up a closed area on the sphere. It is indicated by dark gray in Figure 4. The symmetrical area (with respect to the origin) is another area mapping to the one-transition elementary step functions of type T2: {(1 − *x*_1_(λ; λ_1_)): λ_1_ ∈ [455.4 ; 664]}. It is shown in Figure 4 in light gray.

All the remaining points on the sphere (marked with blue, green, or red in Figure 4) represent **k**s that determine optimal spectral reflectances with two or more wavelength transitions. Each distinct **k** from this area defines a different optimal reflectance. Clearly, if two **k**s determine planes each of which intersects the spectral curve at two (or more) identical points then these planes (thus **k**s) coincide.

Figure 4, therefore, represents a graph of the function *N*(**k**), where *N*(**k**) is the number of transition wavelengths of the optimal reflectance induced by **k**. The number of the transition wavelengths is represented with colors as follows: purple, 0; gray, 1; blue, 2; green, 3; red, 4.

### λ-Parameterization

A second approach to parametrizing the optimal reflectances is to parametrize them in terms of their wavelength transitions, specifically pairs of transition wavelengths determined as follows. First, note that each **k** determines a plane in the color signal space via Equation 8, and if **k** belongs to the blue, green or red areas in Figure 4 then the plane will intersect the spectral curve at a minimum of two distinct points. Consider the plane intersecting the spectral curve at points $\vec{C}\left(\lambda_{1}\right)=\left(s_{1}\left(\lambda_{1}\right),s_{2}\left(\lambda_{1}\right),s_{3}\left(\lambda_{1}\right)\right)$ and $\vec{C}\left(\lambda_{2}\right)=\left(s_{1}\left(\lambda_{2}\right),s_{2}\left(\lambda_{2}\right),s_{3}\left(\lambda_{2}\right)\right)$. Because the plane includes the origin, these points uniquely specify a plane in color-signal space. Its equation is

(23) $$\left|\begin{array}{ccc} z_{1} & z_{2} & z_{3}\\ s_{1}\left(\lambda_{1}\right) & s_{2}\left(\lambda_{1}\right) & s_{3}\left(\lambda_{1}\right)\\ s_{1}\left(\lambda_{2}\right) & s_{2}\left(\lambda_{2}\right) & s_{3}\left(\lambda_{2}\right) \end{array}\right|=0.$$

As in the case of Equation 21, the coefficients *k*_1_, *k*_2_, and *k*_3_ (see Equation 8) can be derived from (Equation 23) as functions of the color signal coordinates of the points on the spectral curve specified by wavelengths λ_1_ and λ_2_:

(24) $$k_{i}\left(\lambda_{1},\lambda_{2}\right)={\left(-1\right)}^{i-1}\left|\begin{array}{cc} s_{p}\left(\lambda_{1}\right) & s_{q}\left(\lambda_{1}\right)\\ s_{p}\left(\lambda_{2}\right) & s_{q}\left(\lambda_{2}\right) \end{array}\right|,$$

with *p*, *q* defined as in Equation 22.

Although this plane may intersect the spectral curve at other points as well, it is, nonetheless, uniquely determined by λ_1_ and λ_2_ because there is only one plane through the origin in the color signal space containing points $\vec{C}\left(\lambda_{1}\right)$ and $\vec{C}\left(\lambda_{2}\right)$. Therefore, although the optimal reflectance corresponding to this plane might have more than two wavelength transitions, it is completely specified by the transition wavelengths λ_1_ and λ_2_. As a result, these two transition wavelengths can be used to designate this optimal reflectance.

There will exist, however, some pairs of transition wavelengths that designate the same optimal reflectance. For example, consider a pair of points, $\vec{C}\left(\lambda_{1}\right)$ and $\vec{C}\left(\lambda_{2}\right)$, that together determine a plane intersecting the spectral curve at some other point, $\vec{C}\left(\lambda_{3}\right)$. In this case, the three pairs—(λ_1_, λ_2_), (λ_2_, λ_3_), and (λ_1_, λ_3_)—all will designate the same optimal reflectance. Thus, all three are possible choices as the designator for that optimal reflectance.

Because parameterization implies bijectivity, multiple designators for the same optimal reflectance must be eliminated. As shown in Appendix A, a subset of transition wavelength pairs can be singled out such that each different pair of transition wavelengths from the subset uniquely specifies an optimal reflectance. Hence, every optimal reflectance can be uniquely designated by a single pair of transition wavelengths. These wavelengths can be thought of as two parameters in terms of which all the optimal reflectances are uniquely specified. It will be referred to as the λ-parameterization.

For the sake of generality, one can formally designate an optimal reflectance having *n* < 2 transitions by imposing some constraints on transition wavelengths λ_1_ and λ_2_. Indeed, formally the perfect reflector can be considered as an elementary step function of degree 2 and type T1 with transition wavelengths λ_min _ and λ_max _ (i.e., *x*_2_(λ; λ_min _, λ_max _)). The perfect absorber, which is of type T2, will be 1 − *x*_2_(λ; λ_min _, λ_max _). Likewise, an elementary step function of degree 1 and type T1 with transition wavelength λ_1_ can be designated as *x*_2_(λ; λ_1_, λ_max _)), and that of type T2 as 1 − *x*_2_(λ; λ_1_, λ_max _).

### The relationship between the **k**- and λ-parameterization

When **k** is fixed then *g* in Equation 7 is a function of λ alone. In general, *g* can be considered as a function *g*(λ, **k**) of both λ and **k**. To explore the relationship between the **k**-parameterization and the λ-parameterization, consider Equation 9 as defining an implicit functional relationship between λ and **k**. Let (λ_0_, **k**_0_) satisfy Equation 9, i.e., *g*(λ_0_, **k**_0_) = 0. According to the implicit function theorem, Equation 9 defines λ as a function of variables *k*_1_, *k*_2_, *k*_3_ in a neighborhood of point (λ_0_, **k**_0_) if the partial derivative

(25) $$g_{\lambda }^{'}=k_{1}s_{1}^{'}\left(\lambda \right)+k_{2}s_{2}^{'}\left(\lambda \right)+k_{3}s_{3}^{'}\left(\lambda \right)$$

is nonzero at (λ_0_, **k**_0_).

**Figure 5.**
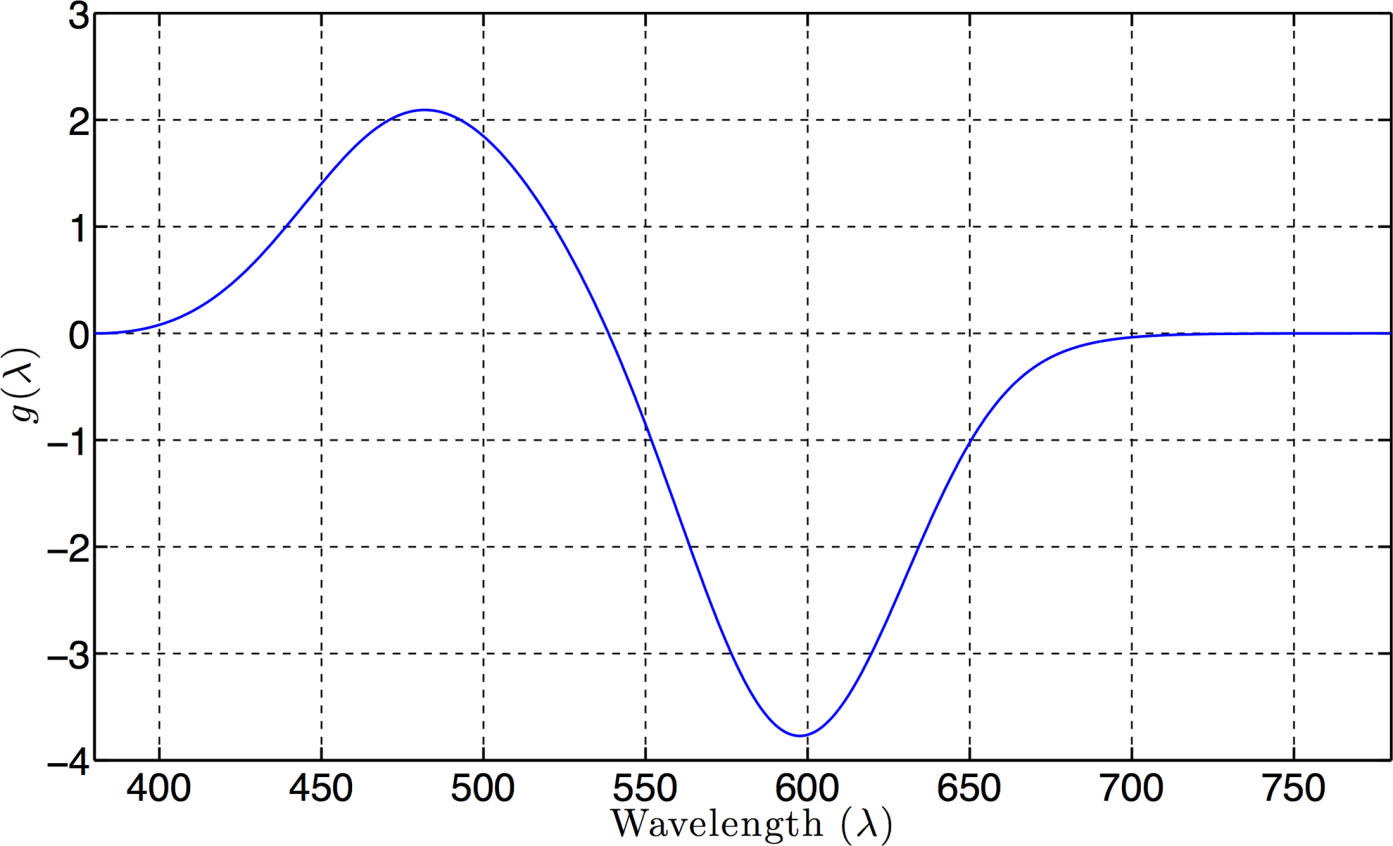
Example plot of *g*(λ) for **k** = (0.0052, 0.5953, −0.8035). In this case $g_{\lambda }^{'}=0$ at λ = 480 nm and λ = 598 nm.

When $g_{\lambda }^{'}\left(\lambda_{0},k_{0}\right)=0$ then *g* as a function of λ has either an extremum at λ_0_ (Figure 5) or an inflection point. If a **k**_0_ determines a plane tangent to the spectral curve at $\vec{C}\left(\lambda_{0}\right),\text{then}\;g\left(\lambda \right)$ must have a local minimum at λ_0_, with the graph of *g* just touching the horizontal coordinate axis at λ_0_. Such a case is singular in the sense that a small change in **k**_0_ causes either the emergence of two roots (instead of the one at λ_0_) close to λ_0_, or the disappearance of the root at, or in the vicinity of, λ_0_. It follows that the point corresponding to **k**_0_ on the **k**-sphere in Figure 4 lies on the border separating areas that differ in the number of wavelength transitions. Specifically, if for this particular **k**_0_ there are no roots other than λ_0_, then **k**_0_ lies on the border of the purple/blue regions (dark purple/blue or light purple/blue for reflectance functions of type T1 and T2, respectively) in Figure 4, separating the zero- and two-transitions areas. If there is just one additional root then it is on the border separating one of the two one-transition areas (dark/light gray) from the three-transition areas (dark/light green). Finally, it might separate the two- and four-transition areas, corresponding to the dark/light blue and dark/light red regions, respectively.

For all other points (λ_0_, **k**_0_) such that $g_{\lambda }^{'}\left(\lambda_{0},k_{0}\right)\neq 0$, λ is a smooth function of **k**, its partial derivatives being

(26) $$\frac{\partial \lambda }{\partial k_{i}}=-\frac{s_{i}\left(\lambda \right)}{k_{1}s_{1}^{'}\left(\lambda \right)+k_{2}s_{2}^{'}\left(\lambda \right)+k_{3}s_{3}^{'}\left(\lambda \right)}.$$

Likewise, each *k*_*i*_ is a smooth function of λ with partial derivative

(27) $$\frac{\partial k_{i}}{\partial \lambda }=-\frac{k_{1}s_{1}^{'}\left(\lambda \right)+k_{2}s_{2}^{'}\left(\lambda \right)+k_{3}s_{3}^{'}\left(\lambda \right)}{s_{i}\left(\lambda \right)}.$$

Last, we determine a condition for the existence of a smooth one-to-one correspondence between the **k**- and λ-parameterizations. Let us fix one of the three *k*s, say *k*_3_ = 1. This constraint is more convenient to use here than that of Equation 20. Of the transition wavelengths satisfying Equation 9, choose two adjacent ones λ_1_ and λ_2_ and consider each as a function of *k*_1_ and *k*_2_. The change of parameterization from *k*_1_ and *k*_2_ to λ_1_ and λ_2_ (and back) will be smooth if the Jacobian matrix

(28) $$\left(\begin{array}{cc} \frac{\partial \lambda_{1}}{\partial k_{1}} & \frac{\partial \lambda_{1}}{\partial k_{2}}\\ \frac{\partial \lambda_{2}}{\partial k_{1}} & \frac{\partial \lambda_{2}}{\partial k_{2}} \end{array}\right)$$

is invertible, i.e., if its determinant is not zero. In light of (Equation 26) and assuming that $k_{1}s_{1}^{'}\left(\lambda \right)+k_{2}s_{2}^{'}\left(\lambda \right)+s_{3}^{'}\left(\lambda \right)\neq 0$ for λ_1_ and λ_2_, the determinant of matrix (Equation 28) is not equal to zero if and only if the following determinant is not zero as well:

(29) $$\left|\begin{array}{cc} s_{1}\left(\lambda_{1}\right) & s_{1}\left(\lambda_{2}\right)\\ s_{2}\left(\lambda_{1}\right) & s_{2}\left(\lambda_{2}\right) \end{array}\right|\neq 0.$$

Because the choice of the pair of *k*s is arbitrary, this inequality needs to hold for any pair of the spectral sensitivity functions. Therefore, the correspondence between the **k**- and λ-parameterizations within an area of the same number (two or more) of wavelength transitions will be smooth when the determinant (Equation 29) is not zero for all three pairs of the spectral sensitivity functions, as is the case for the cone photopigments (Figure 1).

### αδλ -Parameterization

Let us consider the advantages of one more parameterization that is a particular case of the more general parameterization proposed previously by Logvinenko (2009). Specifically, for any spectral reflectance function *x*(λ) producing color signal (φ_1_(*x*), φ_2_(*x*), φ_3_(*x*)) under illuminant *I*(λ) (see Equation 1), there exists a function of the form

(30) $$\begin{array}{c} \left(x_{0.5}\left(\lambda \right)+\alpha \left(x_{2}\left(\lambda ;\lambda_{1},\lambda_{2}\right)-x_{0.5}\left(\lambda \right)\right)\right),\; \end{array}$$

where *x*_2_(λ; λ_1_, λ_2_) is the elementary step function of degree 2 of type T1 (Equation 17) with the transition wavelengths λ_1_ and λ_2_, *x*_0.5_(λ) stands for the flat gray, and α is a real number, such that the following equalities hold:

(31) $$\begin{array}{ccc} & & \;\int_{\lambda_{\min}}^{\lambda_{\max}}\left(x_{0.5}\left(\lambda \right)+\alpha \left(x_{2}\left(\lambda ;\lambda_{1},\lambda_{2}\right)-x_{0.5}\left(\lambda \right)\right)\right)I\left(\lambda \right)s_{i}\left(\lambda \right)d\lambda \;\\ & & \;\;=\varphi_{i}\left(x\right),\;\left(i=1,2,3\right). \end{array}$$

Indeed, given the three numbers φ_1_(*x*), φ_2_(*x*), and φ_3_(*x*), the simultaneous (Equation 31) can be resolved with respect to the three unknowns, λ_1_, λ_2_, and α.

When |α| ⩽ 1, Equation 30 can be interpreted as describing a reflectance function. It is one of the infinite number of the other reflectance functions evoking the color signal (φ_1_(*x*), φ_2_(*x*), φ_3_(*x*)). It can be considered as a representative of the whole class of such functions, the numbers λ_1_, λ_2_, and α being thought of as the parameters characterizing that class (of metameric reflectances).

For a color signal lying on the boundary of the object-color solid, Equation 31 yields |α| ⩾ 1. If |α| = 1, Equation 30 becomes either *x*_2_(λ; λ_1_, λ_2_) or 1 − *x*_2_(λ; λ_1_, λ_2_), depending on the sign of α. In other words, Equation 30 then defines an optimal reflectance with λ_1_ and λ_2_ forming its λ-parameterization. For those color signals on the boundary that are produced by elementary step functions with more than two transition wavelengths, the solution to Equation 31 always results in |α| > 1. Although, the expression in Equation 30 with |α| > 1 cannot be physically implemented as a reflectance function, it can be included as an improper reflectance function (Logvinenko, 2009; Logvinenko & Levin, 2023). In other words, the solutions λ_1_, λ_2_, and α to Equation 31 can be considered as the parameters of an optimal reflectance (corresponding to the color signal in question) even though |α| may exceed 1.

As argued elsewhere (Logvinenko, 2009; Logvinenko & Levin, 2023), it can often be more convenient to convert (λ_1_, λ_2_) to a new set of parameters as follows. Specifically, for an optimal reflectance, *x*_2_(λ; λ_1_, λ_2_) of type T1, define its central wavelength, λ_*c*_ = (λ_1_ + λ_2_)/2, and spectral bandwidth, δ = |λ_1_ − λ_2_|. The reflectance *x*_2_(λ; λ_1_, λ_2_) expressed in terms of its central wavelength and spectral bandwidth will be denoted as *x*_2_(λ; λ_*c*_, δ). For a general definition that encompasses both the type T1 and type T2 cases, see that provided by Equations 13 through 16) in Logvinenko (2009). As shown there, each optimal reflectance is uniquely determined by these three positive numbers, α, δ, and λ_*c*_. These numbers will be referred to as the αδλ-parametrization of optimal reflectances. Adopting the terminology proposed previously (Logvinenko, 2009; Logvinenko and Levin, 2023), we refer to (*x*_0.5_(λ) + α(*x*_2_(λ; λ_*c*_, δ) − *x*_0.5_(λ))) as the *rectangular metamer* of *x*(λ).

The αδλ-parameterization proves to be more natural and convenient than either the λ- or **k**-parameterizations. First, because central wavelength and spectral bandwidth uniquely determine a direction in the color signal space; whereas, the transition wavelengths determine two different directions, one per type, in color signal space. Second, the form of the corresponding optimal reflectance is intuitively easy to deduce from the central wavelength and spectral bandwidth (especially, as compared with the **k**-parameterization). Third, the proximity between the components of two pairs (δ, λ_*c*_) and ($\delta^{'},\lambda_{c}^{'}$) is, in fact, the proximity between the corresponding optimal reflectances (and well correlated with the proximity between the color signals produced by them). In comparison, proximity in **k**-space is highly nonlinearly related to the proximity of the corresponding optimal reflectances. Finally, central wavelength and spectral bandwidth are closely related to such color attributes as hue and blackness/whiteness, respectively (Logvinenko, 2009; Logvinenko & Levin, 2023). Moreover, the parameter α correlates well with the purity of the object color invoked by the rectangular metamer with that α. Such a good correlation of the parameters α, δ, and λ_*c*_ with the perceptual attributes of the object color provides a good reason to use them as color descriptors (e.g., Mirzaei & Funt, 2015a); and to use the entire set (including improper ones) of rectangular metamers as defining the object-color atlas (Logvinenko, 2009; Logvinenko & Levin, 2023)

## Evaluating the trichromatic object-color solid

To get a general idea of the shape of the object-color solid, it suffices to generate a very large sample of optimal reflectances, calculate their corresponding color signals, and then plot them in the color signal space. It seems natural to think that such a plot will sketch the outline of the object-color solid. However, the question is as to how to carry out a sampling of the optimal reflectances that will result in a representative sample in the color signal space, that is, a sample of points in the object-color-solid boundary that gives an accurate idea of the object-color-solid’s shape. It is clear that such sampling should be carried out in parametric form, that is, as sampling in terms of either the **k**- or λ-parameterizations. From the computational point of view, it is easy to generate an optimal reflectance sample using the **k**-parameterization. However, as shown below, a homogeneous sample in **k**-space brings about a sample of the points in the object-color-solid boundary that is very far from homogeneous. This seriously narrows the possibilities of using this method, making it suitable only for constructing the first, very rough sketch.

### Sketching the object-color solid via **k**- and λ-parameterizations

To carry out **k**-parameterization we resort to reparameterization in terms of spherical polar coordinates. In the view of the constraint expressed in Equation 20 the **k**-parameterization is, actually, in terms of a unit sphere. Hence, we introduce spherical polar coordinates β and γ, with γ within (0, 2π), corresponding to the azimuthal angle, and β within (0, π), corresponding to the zenith angle, and generate a uniform sample of the polar angles β and γ. Then, for each sample (β, γ) (i.e., for each **k**), the transition wavelengths (i.e., zero-crossings for *g*(λ) in Equation 7) were calculated. These define an optimal reflectance. Each optimal reflectance *x*_*opt*_(λ) determines a point Φ(*x*_*opt*_) on the object-color-solid boundary to which it maps. This point is found by substituting *x*_*opt*_(λ) into Equation 1 and computing the three components of Φ(*x*_*opt*_).

**Figure 6.**
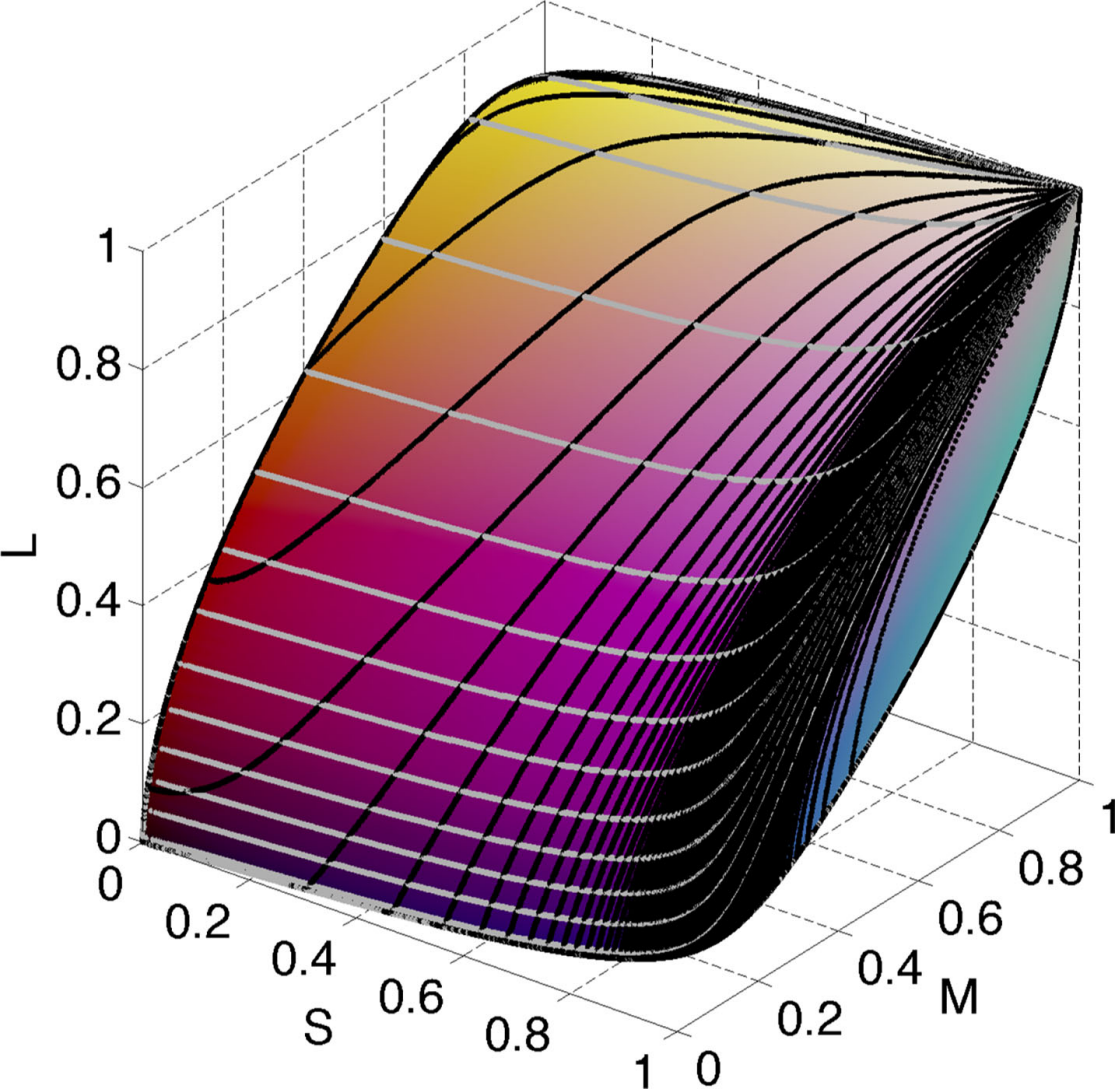
The object-color solid under the equal-energy illuminant in *SML* coordinates. The gray and black contours are, respectively, those of constant γ, the azimuthal angle, and β, the zenith angle, in the spherical coordinate representation of **k** space. The right-hand part of the plot is black simply because the black contour lines become very tightly packed.

**Figure 7.**
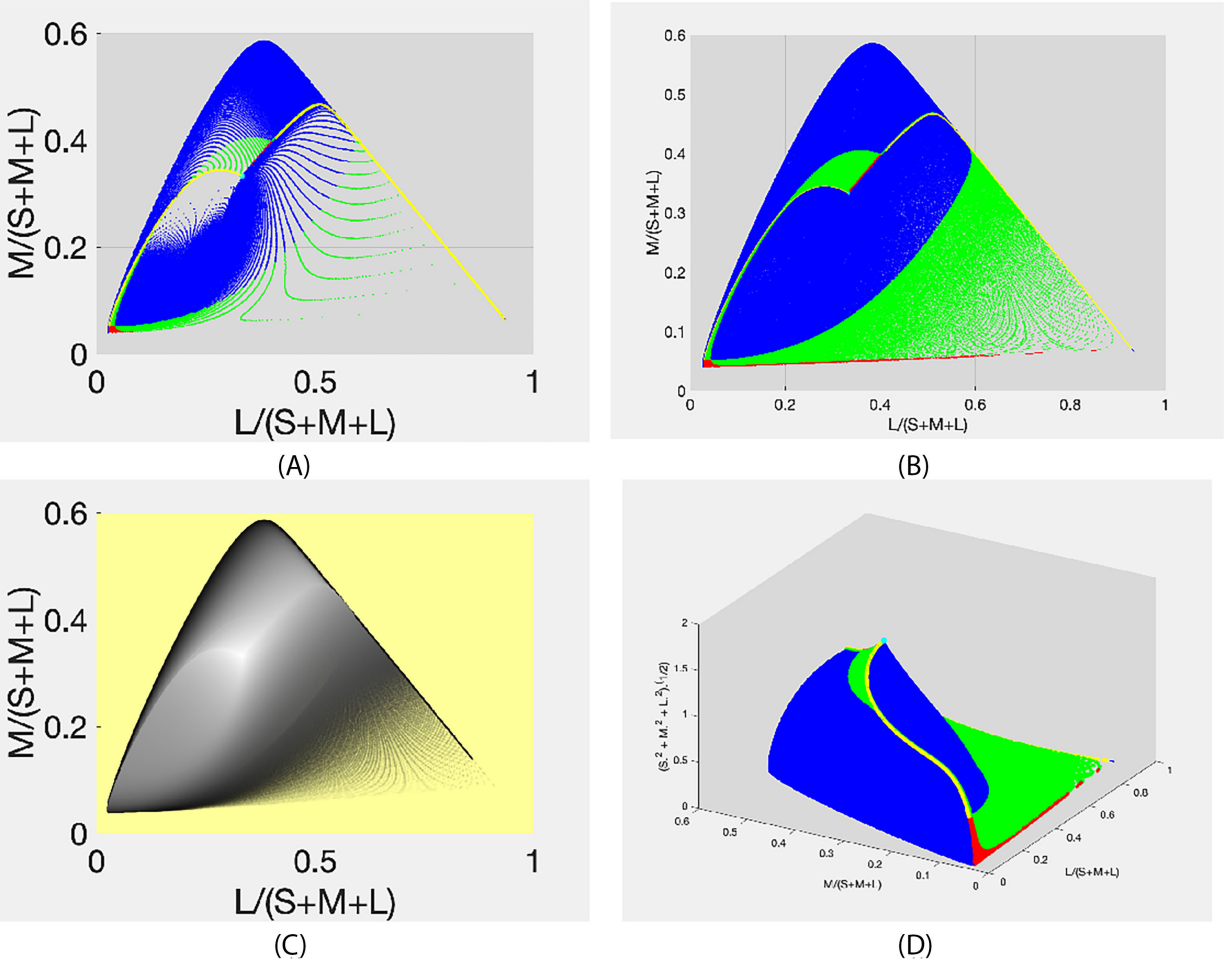
(**A**) Chromaticities of a regular sampling of γ, the azimuthal angle, and β, the zenith angle, in the spherical coordinate representation of **k** space with colors representing the number of transitions (cyan, 0; yellow, 1; blue, 2; green, 3; red, 4) of the corresponding optimal reflectances. (**B**) Chromaticities of a regular λ-sampling for the same number of samples as for (**A**). (**C**) Length of the corresponding λ-sampling SML vector encoded as intensity. (**D**) Rösch’s object-color solid with the vertical axis representing the length of the corresponding SML vector.

Figure 6 presents the object-color solid thus obtained. The solid black and gray contours in Figure 6 correspond to constant values of β and γ, respectively. Note that this regular sampling of β and γ yields a highly irregular grid on the object-color-solid boundary. In other words, a regularly spaced collection of directions (rays) in **k**-space brings about rather irregularly spaced beam of vectors in *SML* space. This irregularity is easily seen in Figure 7A, where the resulting sample of *SML* vectors is presented as the points in the chromaticity plane (same as in Figure 2) corresponding to these vectors. Although the cluster of the chromaticity points in this graph results from a homogeneous sample of the polar angles (qualitatively similar results are found when using geodesic sampling), there are vast areas in the chromaticity plane void of markers despite a rather dense **k**-sampling resulting in 1,288,417 **k**s. This alone is enough to undermine the use of **k**-sampling as a practical way of sketching the object-color solid; however, that is not the only reason. As can be seen from Figure 4, many of the **k**-samples generate either zero- or one-transition reflectances. Specifically, for this sample of polar angles defining a set of **k**-samples the zero-, one-, two-, three-, and four-transition reflectances arise from 27.2%, 55.1%, 17.2%, 0.42%, and 0.008% of the **k**-samples, respectively. Since the chromaticity loci corresponding to the zero- and one-transition reflectances comprise the poles and a curve, that is, occupy a zero-area fragment of the chromaticity diagram, only 18% of the **k**-samples are actually mapped onto the vast majority of the chromaticity gamut. Given the extremely nonlinear relationship between the rays in **k**-space and vectors in *SML* space, we must conclude that **k**-sampling is highly ineffective when using it for evaluating the object-color-solid boundary.

In contrast, homogeneous λ-sampling (for details, see Appendix A) produces a much more homogeneous cluster of chromaticity points as is clearly visible if we look at Figure 7B where the results of λ-sampling are presented in the same way as those of **k**-sampling in Figure 7A. Figure 7B presents the chromaticities resulting from the reflectances generated by homogeneous λ-sampling at a step size of 0.2221 nm. With the number of λ-samples being as large as the number of **k**-samples the pattern in Figure 7B is much denser than in Figure 7A, although it too is not completely even.

Generally, the advantage of λ-sampling over **k**-sampling reveals itself in the following: i) there are no large areas void of chromaticity points (as in Figure 7A); ii) the pattern of chromaticity points effectively covers the entire chromaticity gamut; and last but not least; iii) λ–sampling provides a one-to-one correspondence between the set of parameters and the resulting set of chromaticity points (for details see Appendix A). Therefore, we are in full control of the density of the resultant cluster of the chromaticity points. Hence, in terms of becoming familiar with the general shape of the object-color solid, we recommend using λ-sampling rather than **k**-sampling.

Figure 7B can be made even more informative by associating with each chromaticity point the distance from the origin to its corresponding point (*SML*) on the object-color-solid boundary. In Figure 7C, the greyness of the markers encodes this distance. In Figure 7D it is encoded as the z-value. In other words, what is presented in Figure 7D is the graph of the function *z*(*x*, *y*) where *x* = *L*/(*S* + *M* + *L*), *y* = *M*/(*S* + *M* + *L*)), and $z=\sqrt{\left(S^{2}+M^{2}+L^{2}\right)}$. This volume is referred to by Wyszecki and Stiles (1982) as the Rösch color solid. They are distinguishing it from a volume in the *SML* space like that depicted in Figure 6, which they term the Luther-Nyberg color solid. Figure 7D, therefore, presents the Rösch object-color solid obtained using the homogeneous λ-parameterization.

**Figure 8.**
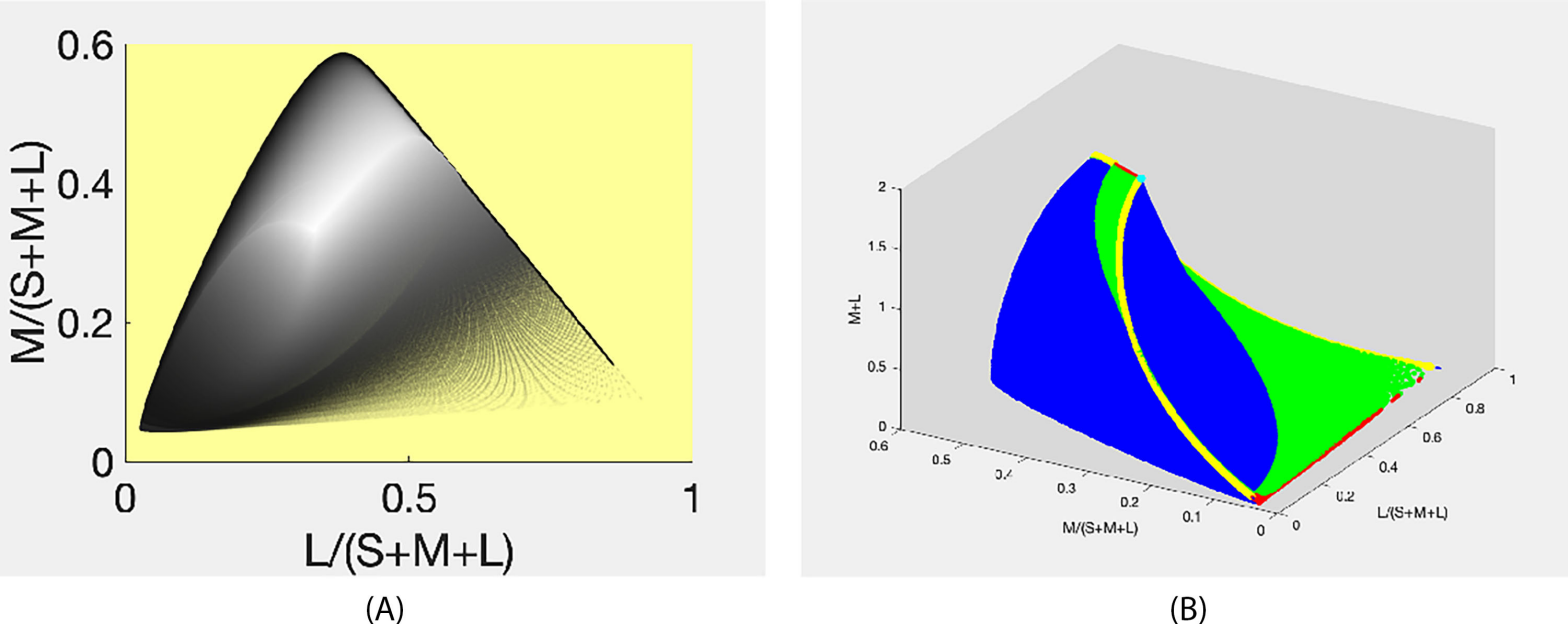
(**A**) Luminance (M + L) of the corresponding λ-sampling SML vector encoded as intensity; and (**B**) The Rosch object-color solid with the vertical axis representing M+L. Colors indicate the number of transitions (cyan 0; yellow 1; blue 2; green 3; red 4).

Figures 7C and 8A show the sum *M* + *L* plotted in terms of the greyness of the markers. By redrawing Figure 7D and plotting *M* + *L* along the *z*-axis as in Figure 8B, we get an idea of the maximal luminance achievable for a given direction (from the origin) in the object-color solid. Indeed, *M* + *L* is generally believed to serve as a good estimate of luminance (Lennie, Pokorny, & Smith, 1993). Here it is appropriate to remember that it was this problem, namely, that of finding the brightest surface having a given chromaticity, which prompted Schrödinger to study the object-color solid. Whereas Figures 7D and 8B give a general idea of what the answer to this question might be, determining the exact answer for a specific chromaticity requires finding its corresponding optimal reflectance. We refer to the problem of finding this optimal reflectance as *Schrödinger’s problem*.

**Figure 9.**
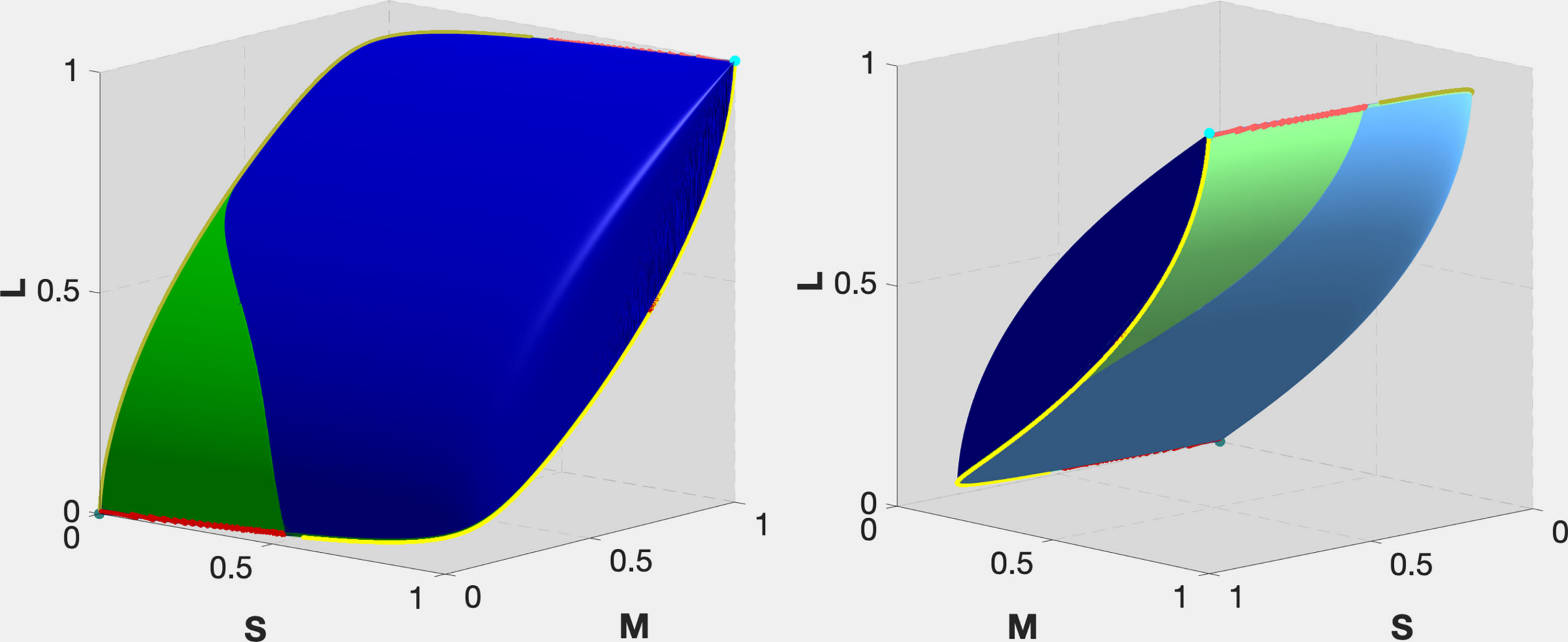
Two sides of the object-color solid under the equal-energy illuminant in *SML* coordinates. The plot on the right depicts the side opposite to that on the left. Because of the central symmetry of the object-color solid, the near side (left) looks symmetrical to the far side (right). The coloring indicates the number of transition wavelengths of the corresponding optimal reflectance (cyan, 0; yellow, 1; blue, 2; green, 3; red, 4). Darker colors indicate type T1 optimal stimuli, lighter ones type T2.

It is noteworthy that a significant part of the chromaticity gamut in Figure 7B is colored green. In other words, the chromaticity area corresponding to the three-transition reflectances is larger than one would think when comparing the green and blue areas in Figure 9. It is curious that this difference is hidden when the object-color solid as such is observed (see Figure 9). Figure 9 presents the object-color solid obtained by using the λ-parameterization. The areas of different numbers of transition wavelengths are marked with different colors in the same way as in Figure 7. As can be seen, the object-color solid boundary consists of two symmetrical parts, which differ in type. Each part contains a curve, a portion of which is in yellow (one-transition-wavelength reflectances) with a second portion in red (four-transition-wavelength reflectances), that begins at one pole and ends at the other. Let us call these two lines the main meridians. Together they form a close loop that delimits these symmetrical halves of the object-color solid boundary. (Note, however, that each half in question does not have a closed boundary. As a geometrical analog, consider a square two sides of which belong to it, and the other two do not.)

Before moving on to Schrödinger’s problem, a few words about the αδλ-parameterization in the present context. It cannot be used for sketching the object-color solid since it implies solving Schrödinger’s problem. This is the case because the αδλ-parameterization requires the set of optimal reflectances undergoing the parameterization to be specified in advance. True, one can produce a uniform sample for δ and λ_*c*_; however, to find out what α corresponds to a particular pair (δ, λ_*c*_), one has to i) determine the direction in the color signal space that is determined by this pair; ii) determine the optimal reflectance corresponding to this direction (Schrödinger’s problem); iii) determine the point in this direction corresponding to this optimal reflectance; and iv) compute α for that point. Certainly, when this optimal reflectance has precisely two wavelength transitions the last two steps can be omitted, because in that case α equals 1.

**Figure 10.**
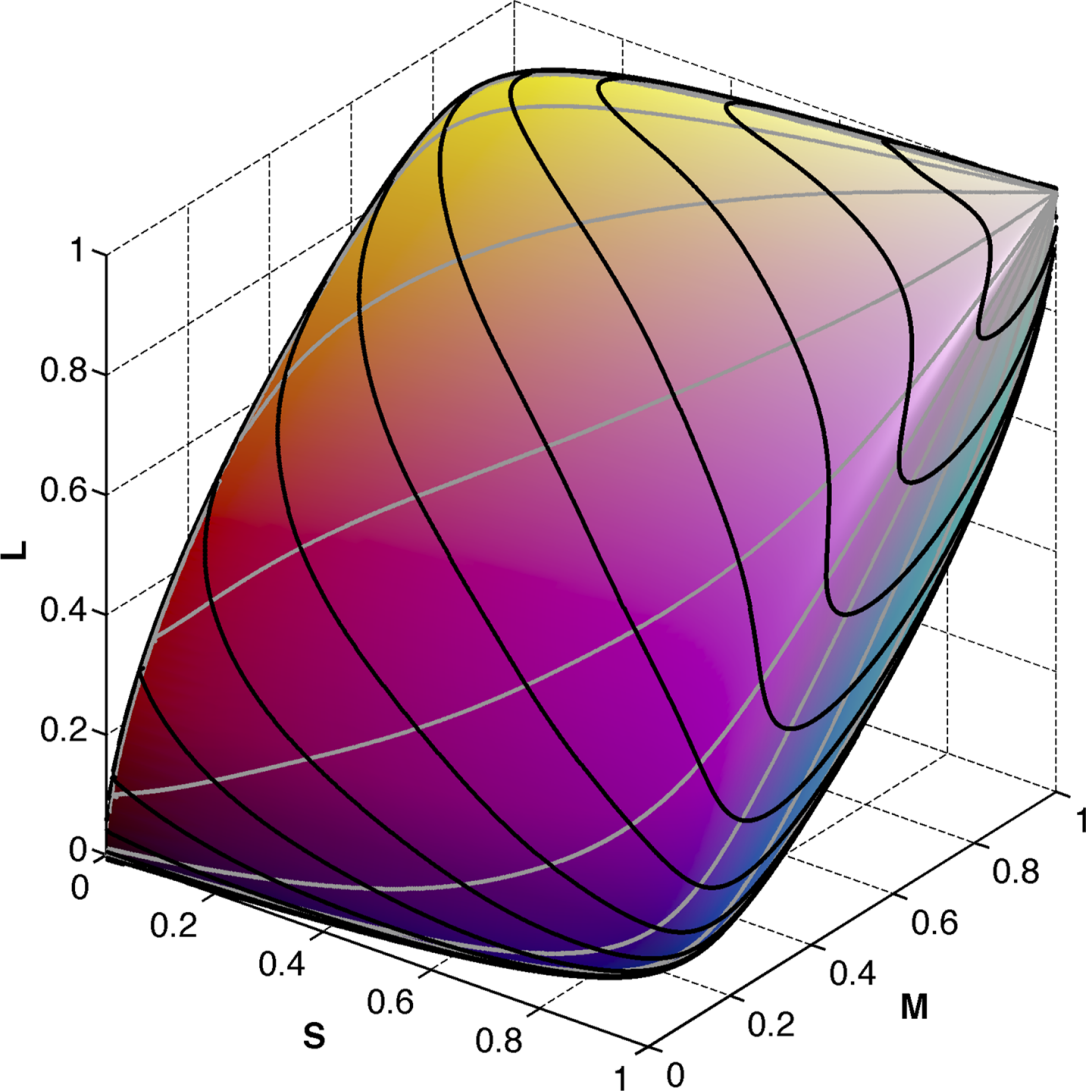
The regular (i.e., two-transition-reflectance-based) object-color solid in *SML* coordinates under the equal-energy illuminant. The contours are those of constant δ (*black*) and constant λ_c_ (*gray*).

As follows from Figures 7 to 9 the number of directions with more than two wavelength transitions is considerable. Still, as mentioned, it is common practice to ignore this fact and simply consider a plot similar to that in Figure 10 as representing the true object-color solid. What is shown in this figure—let us call this volume “the regular object-color solid”—is the plot of all two-transition step functions based on a regular sample of δ and λ_c_. Notice, by the way, that the black and gray grid of contours for fixed δ and λ_c_, respectively, is more uniform than in Figure 6. The regular object-color solid constitutes a volume inscribed within the true object-color solid.

### Identifying the object-color solid by solving Schrödinger’s problem

Each ray in the color-signal space intersects the boundary of the object-color solid just once. Specifically, given the ray determined by the color signal (*S*, *M*, *L*), there exists a unique vector (*S*_0_, *M*_0_, *L*_0_) from the origin, collinear to (*S*, *M*, *L*) and ending at the point of intersection with the boundary. The vector (*S*_0_, *M*_0_, *L*_0_) is determined by the optimal reflectance corresponding to the ray in question. Therefore, establishing the object-color solid equates to the problem of identifying the optimal reflectance corresponding to the direction (ray) determined by any arbitrary (*S*, *M*, *L*). In other words, we have to derive the optimal reflectance *x*_*opt*_(*S*, *M*, *L*) from (*S*, *M*, *L*), and then evaluate the color signal (*S*_0_, *M*_0_, *L*_0_) corresponding to *x*_*opt*_(*S*, *M*, *L*). This is where Schrödinger’s problem lies. Plotting the resulting points (*S*_0_, *M*_0_, *L*_0_) in the color-signal space will identify the object-color-solid boundary (as a cloud of discrete points).

In mathematics, this type of problem is called an inverse problem. Given a color signal $\left(z_{1}^{0},z_{2}^{0},z_{3}^{0}\right)$, find the optimal reflectance *x*_*opt*_(λ) such that $\Phi \left(x_{opt}\right)=\kappa \left(z_{1}^{0},z_{2}^{0},z_{3}^{0}\right)$, where κ is some unknown constant. In other words, find the optimal reflectance *x*_*opt*_(λ) satisfying the following equations:

(32) $$\;\int_{\lambda_{\min}}^{\lambda_{\max}}x_{opt}\left(\lambda \right)I\left(\lambda \right)s_{i}\left(\lambda \right)d\lambda =\kappa z_{i}^{0},\;i=1,2,3.$$

The existence of a solution to such equations becomes clear when the optimal reflectance is expressed in a parametric form, a solution being sought with respect to the parameters. For instance, using the λ-parameterization we arrive at the following equivalent form of Equation 32:

(33) $$\;\int_{\lambda_{\min}}^{\lambda_{\max}}\tilde{x}\left(\lambda ;\lambda_{1},\lambda_{2}\right)I\left(\lambda \right)s_{i}\left(\lambda \right)d\lambda =\kappa z_{i}^{0},\;i=1,2,3,\;$$

where λ_1_ and λ_2_ are the smallest two transition wavelengths of the optimal reflectance $\tilde{x}$.

Being a set of three simultaneous equations with respect to three unknowns (λ_1_, λ_2_ and κ), (Equation 33) have an exact solution for any color signal $\bar{z}{}^{0}=\left(z_{1}^{0},z_{2}^{0},z_{3}^{0}\right)$. However, to find it for some particular $\bar{z}{}^{0}$, one has to resort to numerical methods, which can only bring an approximate solution. That is, the solution can be found only with a certain degree of accuracy. Solving the inverse problem numerically is not an easy task and the topic deserves a separate paper. Below we restrict ourselves to illustrating the nature of the problems that arise both when applying the well-known Newton method for solving (Equation 33) (see Appendix B) as well as when applying some other methods available as part of Matlab’s repertoire.

**Figure A1.**
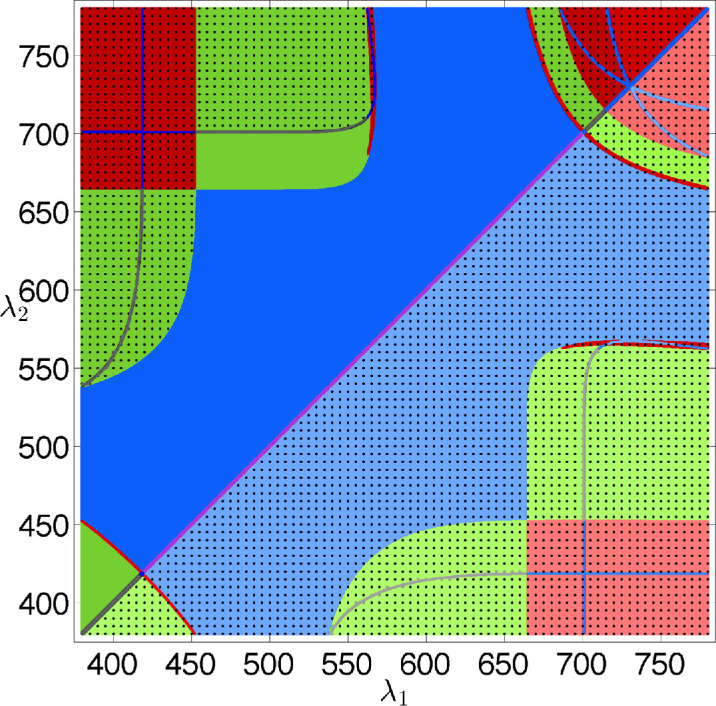
Plot of the number of transitions *N*(λ_1_, λ_2_) for λ_1_ ⩽ λ_2_. The color coding is the same as in Figure 4: dark/light purple for zero transitions, dark/light gray for one transition, dark/light blue for two transitions, dark/light green for three transitions, dark/light red for four transitions. The dark colors correspond to type T1 reflectances, the light colors with type T2. The graph for λ_1_ ⩾ λ_2_ is symmetrical and corresponds to type T2 optimal reflectance functions. The area that is not overlaid with dots defines the admissible region, $\bar{\Lambda^{2}}$, for the λ-parametrization: each different pair of transition wavelengths from this subset specifies a different optimal reflectance function.

A uniform sampling of Λ^2^ (with a 1.5 nm wavelength separation) leads to 35,511 pairs (λ_1_ < λ_2_). Of these, 22,260 pairs turned out to be admissible (see Figure A1 in Appendix A). For these admissible pairs, the corresponding optimal reflectances were evaluated for both types (as described in Appendix A). The resulting 44,520 optimal reflectances will be referred to as the *test reflectances*. For each test reflectance its *SML* coordinates (i.e., the *test color signal*) were computed and then substituted into (Equation 33) as $\bar{z}{}^{0}$. The optimal reflectance obtained by solving (Equation 33) with this $\bar{z}{}^{0}$ will be referred to as the *solved reflectance*. The *SML* coordinates of the solved reflectance will be denoted as the *solved color signal*.

**Figure 11.**
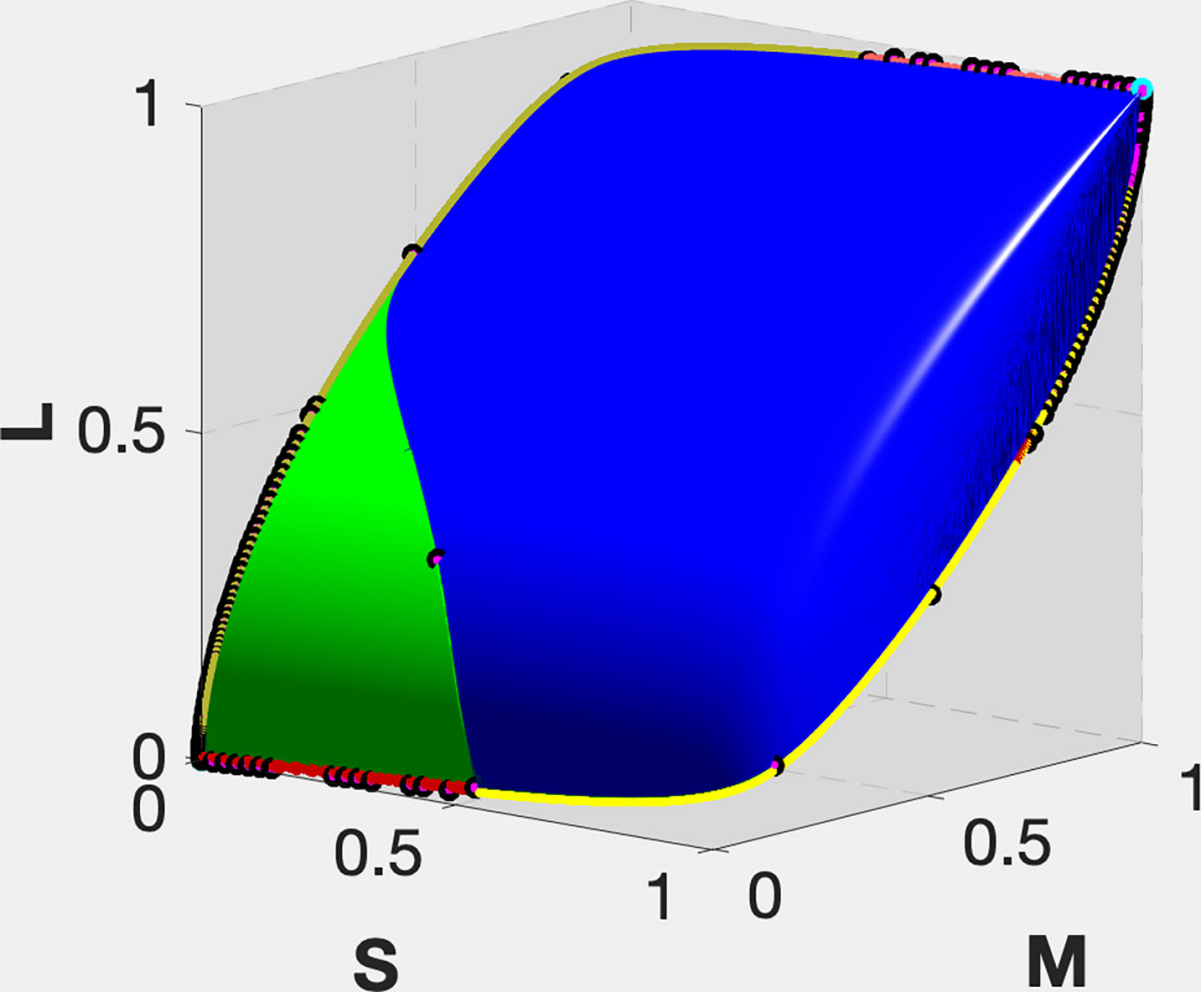
Areas of the object-color solid coded as in Figure 9 with the addition of black dots overlaid, indicating unsolved cases.

**Figure 12.**
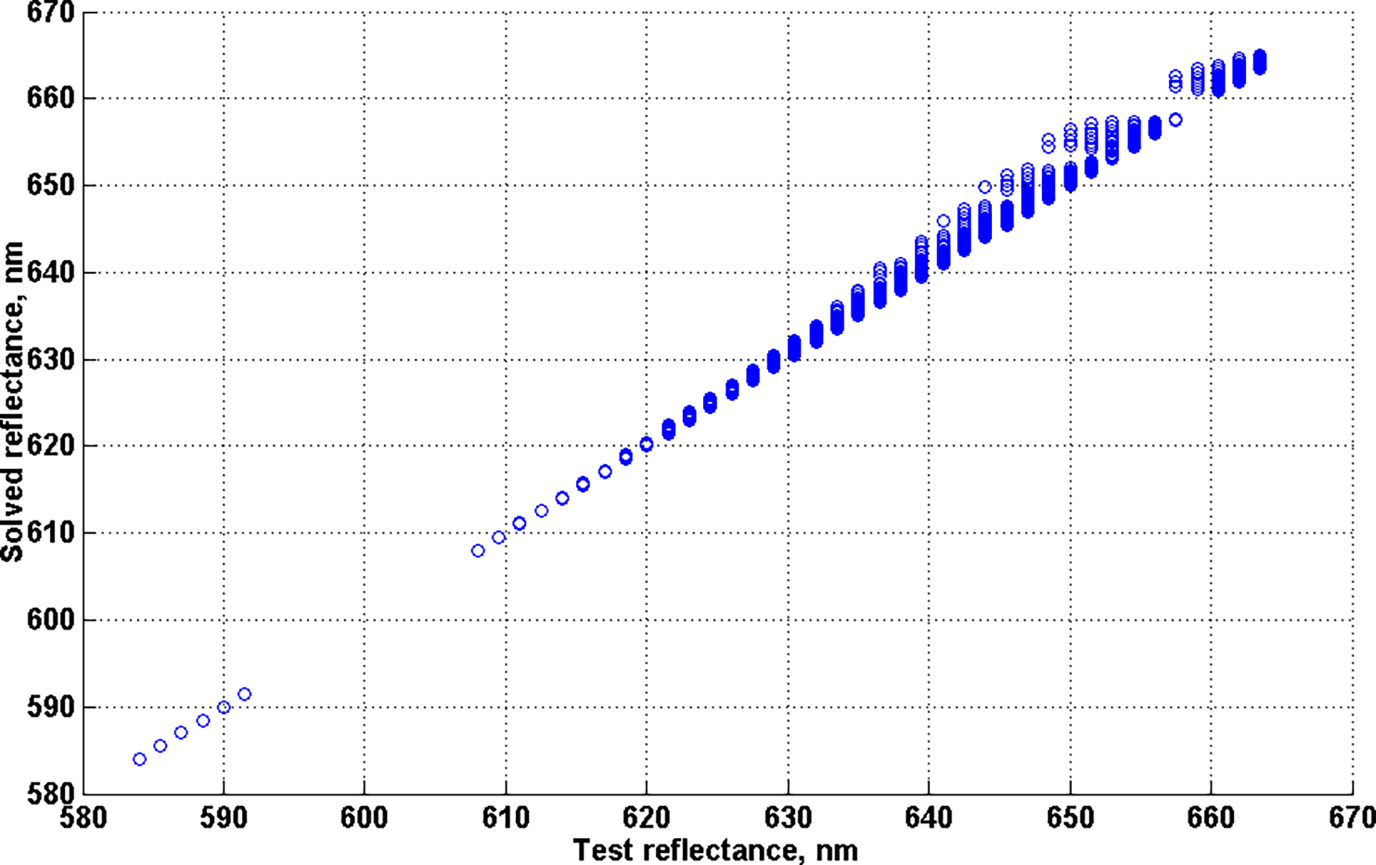
Wavelength of first transition of the solved reflectance vesus wavelength of first transition of the test reflectance.

**Figure 13.**
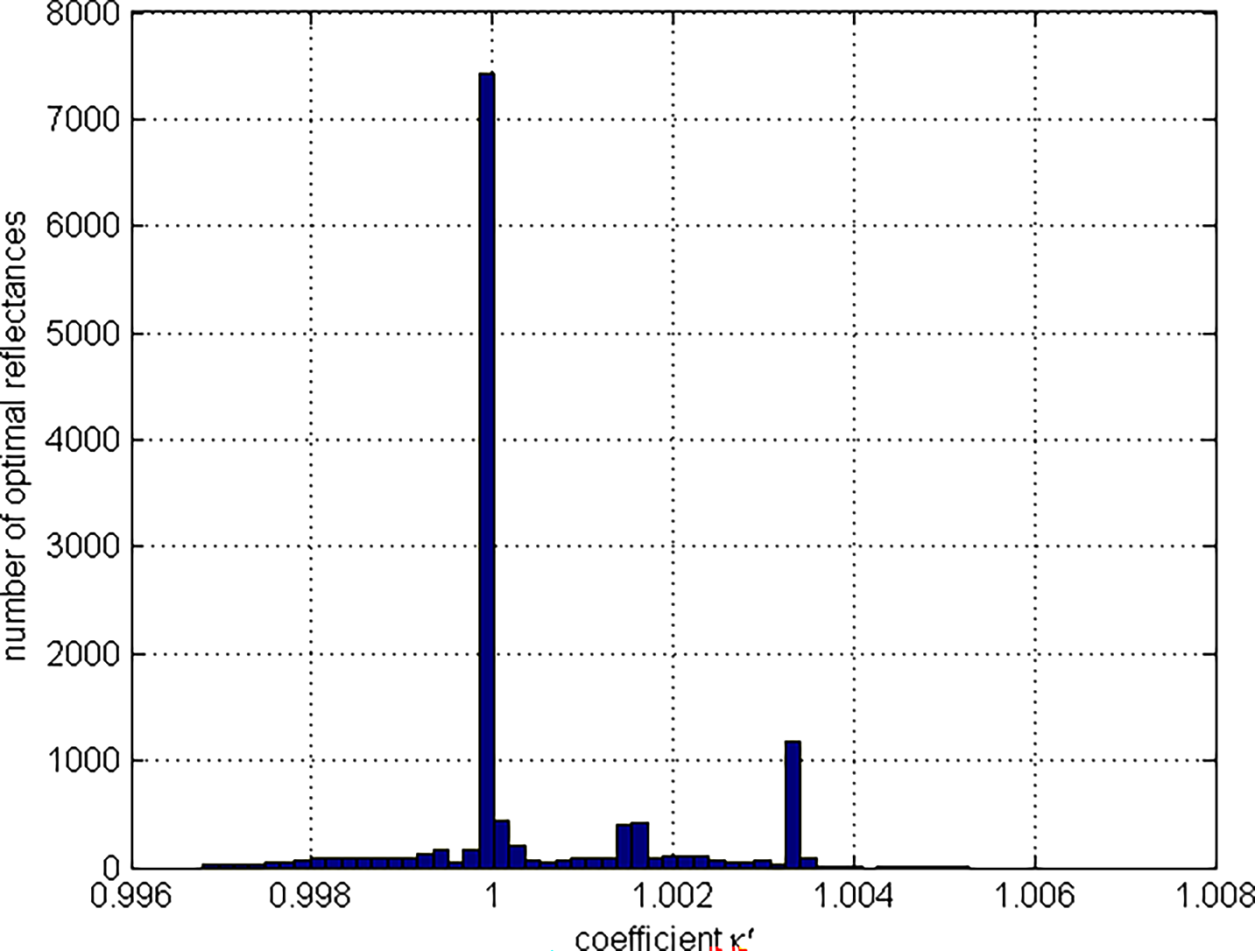
Histogram of the norm ratio $∥\Phi \left(x\left(\kappa \lambda ;\lambda_{1}^{0},\lambda_{2}^{0}\right)\right)∥/∥\left(z_{1}^{0},z_{2}^{0},z_{3}^{0}\right)∥$.

**Figure 14.**
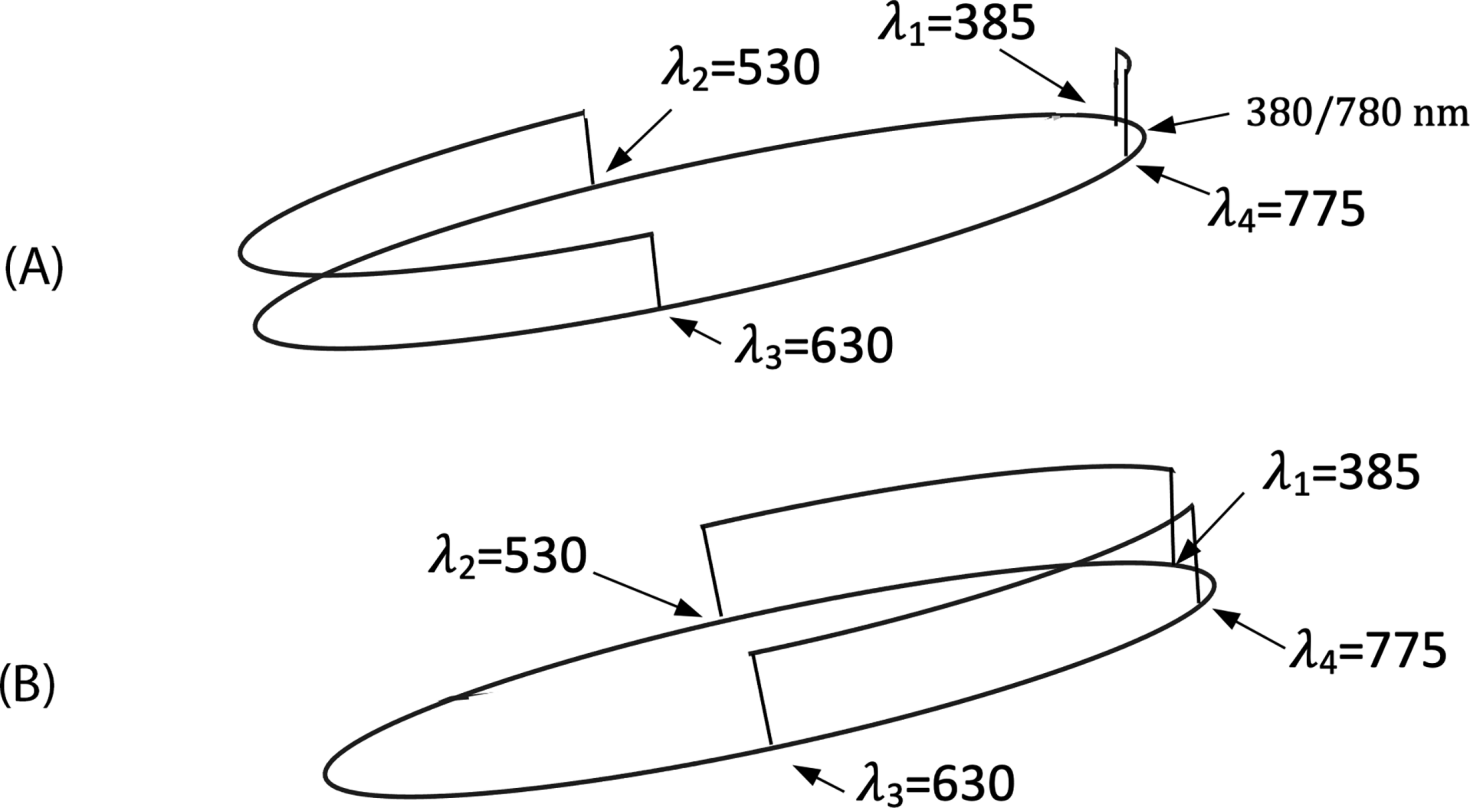
(**A**) A four-transition elementary step function *x*(λ; λ_1_, λ_2_, λ_3_, λ_4_) defined on the spectrum circle; and (**B**) its complementary reflectance $\tilde{x}\left(\lambda ;\lambda_{1},\lambda_{2},\lambda_{3},\lambda_{4}\right)$, which now has two main bumps as discussed in the text.

The median angular difference between the solved and test color signals was found to be 0.0041 arc minutes, the maximum difference being 2.4008 arc minutes. Only 258 (0.58%) solved color signals differ from the test color signals by more than 1 arc minute. The proximity (ideally, coincidence) of color signals is a necessary (but, of course, not sufficient) condition for the solution. We decided to leave out of consideration those cases when the angular difference exceeded 1 arc minute; that is, consider the solved reflectance as an acceptable solution only if the angular difference in question is less than 1 arc minute. Of the remaining 44,262 reflectances (they will be referred to as *admitted reflectances*), 39,580 (89.4%) were of degree 2 (i.e., with two transition wavelengths); 4,654 (10.5%) of degree 3 (i.e., with three transition wavelengths); and 28 (0.06%) of degree 4 (i.e., with four transition wavelengths); all of both types.

We considered the inverse problem (33) to be solved for some direction in the color signal space (i.e., for some test color signal) if i) the solved reflectance is of the same degree (i.e., it has the same number of transition wavelengths) and type as the test reflectance; and ii) the difference between each pair of corresponding transition wavelengths does not exceed 0.1 nm in absolute value. In total, the solution was found for 81.3% of the test reflectances. In particular, the solution was found for 79.1% of the two-transition test reflectances; for 99.96% of the three-transition test reflectances; and for 100% of the four-transition optimal reflectances.

Figure 11 demonstrates how the no-solution cases (marked in black) are distributed over the object-color-solid boundary. It should be noted that, because of the small difference between their *SML* coordinates, many test reflectances map to the same black dot so there are fewer dots than cases.

Relaxing the threshold value from 0.1 nm to 1 nm does not lead to a significant increase in the number of solutions, which in this case only grows to 83.3%. This is because the number of cases in which the test and solved reflectances differ only in degree is not affected by the threshold value. In other words, if the solution is of the right degree, its accuracy (in terms of transition wavelengths) is generally quite high.

As a matter of fact, the vast majority of degree mismatches (96.3%) occur in cases when the test reflectance has two transition wavelengths, whereas the solved reflectance has only one transition wavelength. It should be noted, however, that an elementary step function with one transition wavelength (i.e., of degree 1) can be thought of as a particular case of an elementary step function with two transition wavelengths (i.e., of degree 2), when one of its transition wavelengths is either 380 or 780 nm. This becomes clear if we consider defining the step functions, as Logvinenko (2009) suggests, on the spectrum circle rather than on the spectrum interval. The latter can be achieved by identifying the ends of the spectrum interval and ‘gluing’ them together. On the spectrum circle, step functions having no more than what two wavelength transitions (of both types) have a single rectangular bump (defined by the wavelengths at which the function takes value 1), and functions with three and/or four wavelength transitions have two rectangular bumps, usually, one wider than the other. Hence, elementary step functions with either one or two transition wavelengths (of both types) have the same waveform; namely, a single bump (i.e., where the reflectance function takes 1). The difference between them can be specified by the bump width and the bump position on the spectrum circle. The same is the case for elementary step functions with three and four transition wavelengths (i.e., of degree 3 and 4). Their waveform comprises two bumps, the difference between the elementary step functions being determined by the difference between their respective bumps. Therefore, the genuine difference in kind takes place only when for a test reflectance with two transition wavelengths the solved reflectance is found to have either three or four transition wavelengths. The share of such cases (of the admitted reflectances) turns out to be only 0.38%.

Still, even when such cases are excluded, the proportion of solutions does not increase because the second transition wavelength of the test reflectance in question varies over a wide range (from 663.5 nm to 779 nm), being less than the second transition wavelength of the solved reflectance (i.e., 780 nm) by at least 1 nm. Such discrepancy between the second transition wavelengths is in striking contrast with a fairly accurate match between the first transition wavelengths of the test and solved reflectances (Figure 12). Specifically, the median absolute difference between the first transition wavelengths of the test and solved reflectances is found to be 0.03 nm, the 99th percentile being 4.02 nm. We believe it is all about where the transition wavelengths are in the visible spectrum interval. As a matter of fact, in this particular case, the second transition wavelengths of the test reflectance gravitate towards the long-wave end of the visible spectrum. (It should be said, however, that not every two-transition reflectance, with the second transition wavelength falling into this range, leads to a one-transition solved reflectance, producing such a large error. In particular, for 3,839 two-transition test reflectances satisfying this condition (which is 8.7% of the admitted number of reflectances), solutions were successfully found (with an accuracy of 0.1 nm).

Still, the fact is that even a considerable shift in the transition wavelength in this range of the visible spectrum interval leads to an extremely small effect on the color signal produced by the optimal reflectance. It is worth remembering that we are talking only about the admitted reflectances (the color signals of which differ from the corresponding test color signals by no more than 1 arc minute). In other words, a difference (between the second transition wavelengths) of tens of nanometers does not lead to any noticeable angular difference between the color signals (it remains less than 1 arc minute) provided that this occurs at the long (or short) wavelength end of the visible spectrum interval. This is due to the highly nonlinear relationship between proximities in terms of color signal and those in terms of wavelength.

Such a strong discrepancy between the *SML*-proximity and the wavelength proximity presents a serious problem for solving the inverse problem. It is an issue that has often been overlooked. In particular, in some cases, it makes it almost impossible to solve the inverse problem since the *SML* proximity does not guarantee the wavelength proximity. Specifically, as shown above, despite the closeness of less than 1 arc minute between the test and solved color signals, the difference between the transition wavelengths can be large; the solved reflectance can be of a different degree; and even of a different type.

As to type, there were six cases when the test and solved reflectances were of different type. Consider one of these cases as an example. The test reflectance is of type T1 with two transitions wavelengths: 441.5 and 587 nm. The solved reflectance is of type T2 with three transitions wavelengths: 380.02, 441.5, and 587 nm. The latter comprises two bumps, one of which is virtually identical to the bump of the test reflectance. The second bump is so narrow (0.2 nm wide) that its contribution to the *SML* value is negligible (making the angular difference of 0.01 arc minute).

It is worth noting that a change in type can occur without a change in degree. If one of the transition wavelengths is close to either end of the visible spectrum interval (i.e., to the point on the spectrum circle where the long wavelength end of the spectrum is ‘glued’ to the short wavelength end), then its shift beyond the gluing point will lead to a type change. If the shift is small enough, there will be no significant change in the color signal. This accounts for the appearance of solutions of the wrong type.

Generally speaking, given a test color signal, the type and degree of the corresponding optimal reflectance (i.e., a key component of the solution of the inverse problem for this color signal) can be determined in advance. To do this, one needs to find out the color of the area in Figure 9 to which the direction specified by this signal projects. Note that Figure 9 results from Figure A1 (in Appendix A) by applying the map Φ = (φ_1_, φ_2_, φ_3_) (see Equation 1) to it. How to evaluate the boundaries between the regions (of various colors) in Figure A1 is described in Appendix A. In principle, the search for a solution to the inverse problem should then be carried out in areas of the admissible region $\bar{\Lambda^{2}}$ with the same number of transition wavelengths (i.e., of the same color in Figure A1 in Appendix A). This condition was not enforced in our calculations, which, judging by the results, caused some number of traversals across these boundaries.

In brief, one has to start by calculating in advance the boundaries between these areas of the same degree and type on the object-color solid surface (in terms of *SML* coordinates). Given these boundaries, one can immediately find out the degree of the solved optimal reflectance, and then, using calculations, refine the values of the transition wavelengths, not going beyond them in the process of finding a solution. Note that the algorithm in Appendix B does not imply changing the degree during the course of the solution. Once the initial value is chosen at the beginning, the degree remains the same throughout the solution process.

Last but not least, as mentioned elsewhere in this article, the object-color solid in the region of the black pole is shaped like the color cone, its southern apex is just as sharp. Due to the central symmetry of the object-color solid, the same is the case for the northern apex. Hence, the poles of the object-color solid are not smooth. Also, as Logvinenko and Levin (2023, p. 383) showed, there is a lack of smoothness for points on both meridians. The method described in Appendix B can be used only for smooth points of the object-color solid boundary. Therefore, it is not surprising that such a straightforward method does not work properly for such points. As seen in Figure 11, a fairly significant proportion of black dots (marking cases of no solution) are located along the main meridians, especially near the poles. A more subtle method is required to solve the inverse problem for directions close to the main meridians. Still, whatever the method, there will always be small regions around the meridians that will remain unsolved.

## The two-transition approximation of the trichromatic object-color solid

The regular object-color solid (i.e., the set of color signals induced by a set of reflectances $\kappa X^{'}$ (0 ⩽ κ ⩽ 1) where $X^{'}$ stands for all elementary step functions of degree *m* ⩽ 2) lends itself as a 2-transition approximation of the true object-color solid. In other words, the two-transition approximation to the trichromatic object-color solid is a volume delimited by the image, $\Phi \left(X^{'}\right)$, of the map $\Phi :X^{'}\to R^{3}$, where Φ = (φ_1_, φ_2_, φ_3_). Really, $\Phi \left(X^{'}\right)$ lies in the object-color solid $\Phi \left(X\right)$. Moreover, the boundary of the object-color solid, $\partial \Phi \left(X\right)$, and $\Phi \left(X^{'}\right)$ partially coincide. Specifically, they overlap along those directions (from the origin) in the color signal space for which the corresponding optimal reflectances are the elementary step functions of degree *m* ⩽ 2. For all other directions, the points of $\Phi \left(X^{'}\right)$ lie closer to the black pole of the object-color solid. For, according to the estimates made by Logvinenko (2009), the deviation of the points of $\Phi \left(X^{'}\right)$ from the true object-color solid boundary is rather small, $\Phi \left(X^{'}\right)$ is quite suitable for the role of approximation of $\partial \Phi \left(X\right)$. Because, the use of $\Phi \left(X^{'}\right)$ instead of $\partial \Phi \left(X\right)$ has become a common practice in color research, it is interesting to find out what errors are made in this case. The very fact that committing such errors is not always recognized only adds to the importance of such research.

To quantify the discrepancy between the boundary of $\Phi \left(X\right)$ and $\Phi \left(X^{'}\right)$, one can use elementary step functions of degree *m* ⩽ 2 with a value greater than unity. In other words, allowing for the elementary-step-function amplitude to be greater than one, one can consider a set, $X^{''}$, of elementary step functions of degree *m* ⩽ 2 whose Φ-image will then be exactly the object-color-solid boundary, i.e., $\Phi \left(X^{''}\right)=\partial \Phi \left(X\right)$. In particular, consider an elementary step function, $x\left(\lambda ;\lambda_{1}^{0},\lambda_{2}^{0}\right)$, of degree 2, and its Φ -image: $\Phi \left(x\left(\lambda ;\lambda_{1}^{0},\lambda_{2}^{0}\right)\right)=\left(z_{1}^{0},z_{2}^{0},z_{3}^{0}\right)$. If $\left(z_{1}^{0},z_{2}^{0},z_{3}^{0}\right)$ does not belong to $\partial \Phi \left(X\right)$, one can always find such number κ > 1 that $\Phi \left(\kappa x\left(\lambda ;\lambda_{1}^{0},\lambda_{2}^{0}\right)\right)$ lies in $\partial \Phi \left(X\right)$. Note that κ > 1 corresponds to a theoretical reflectance >100%. The ratio 1/κ can be used to characterize the goodness of the two-transition approximation. Indeed, it is effectively the ratio of the distance from the origin to point $\Phi \left(x\left(\lambda ;\lambda_{1}^{0},\lambda_{2}^{0}\right)\right)$ versus to $\Phi \left(\kappa x\left(\lambda ;\lambda_{1}^{0},\lambda_{2}^{0}\right)\right)$. Therefore, it shows how close $\Phi \left(x\left(\lambda ;\lambda_{1}^{0},\lambda_{2}^{0}\right)\right)$ is to the corresponding boundary point $\Phi \left(\kappa x\left(\lambda ;\lambda_{1}^{0},\lambda_{2}^{0}\right)\right)$.

We evaluated κ for 10,681 three-transition and 7,606 four-transition optimal reflectance functions resulting from an approximately uniform sample of the corresponding three- and four-transition areas in the admissible region $\bar{\Lambda^{2}}$ (the green and red areas in Figure A1 in Appendix A). For every color signal, $\left(z_{1}^{0},z_{2}^{0},z_{3}^{0}\right)$, determined by these 18,287 optimal reflectances, we were seeking a two-transition elementary step function $x\left(\lambda ;\lambda_{1}^{0},\lambda_{2}^{0}\right)$ and a number κ such that $\Phi \left(\kappa x\left(\lambda ;\lambda_{1}^{0},\lambda_{2}^{0}\right)\right)=\left(z_{1}^{0},z_{2}^{0},z_{3}^{0}\right)$ using numerical methods similar to those used when solving the inverse problem in the preceding section. Solutions were found for 17,425 color signals (95.3%). Of these, we took into consideration only those for which the angle between the vectors $\left(z_{1}^{0},z_{2}^{0},z_{3}^{0}\right)$ and $\Phi \left(x\left(\kappa \lambda ;\lambda_{1}^{0},\lambda_{2}^{0}\right)\right)$ (in the color signal space) did not exceed 1 minute of arc. Such cases accounted for three-quarters (13,069) of the total number of solutions. As an estimate of κ, the norm ratio $\kappa^{'}=∥\Phi \left(x\left(\kappa \lambda ;\lambda_{1}^{0},\lambda_{2}^{0}\right)\right)∥/∥\left(z_{1}^{0},z_{2}^{0},z_{3}^{0}\right)∥$ was evaluated. The largest κ′ was found to be 1.133, the smallest being 0.989. The presence of solutions with κ′ less than one, we believe, is a consequence of inevitable computational errors.

Figure 13 shows the relative frequency distribution of the κ′ values over the interval between the 1^*st*^ and 99^*th*^ percentiles (i.e., [0.9968,1.0053]). Although the distribution is evidently skewed toward values greater than 1, its mean (1.0005) does not considerably differ from 1, and its median is actually 1. This indicates that the two-transition approximation is, in fact, very good. Hence, for any practical purpose, the object-color solid boundary can be evaluated by using only two-transition elementary step functions. As our calculations show, the resulting error will be negligibly small (from the practical point of view).

The fact that *SML* coordinates of two-transition reflectances provide a good approximation to the *SML* coordinates of arbitrary *n*-transition optimal reflectances on the object-color-solid boundary does not, however, mean that two-transition elementary step functions can be considered as an appropriate approximation of the optimal reflectances themselves. The idea of such an approximation naturally arises if we consider step functions as defined on the spectrum circle rather than on the spectrum. On the spectrum circle, step functions with three and/or four wavelength transitions have two rectangular bumps, usually, one wider than the other. Moreover, for many optimal stimuli one bump turns out to be a great deal wider than the other. Let us call this wider bump the main bump, and the pairs of wavelengths that define it, the main transition wavelengths. As a matter of fact, the bump of the two-transition approximation (for an optimal reflectance with three and/or four wavelength transitions) is found, quite often, to be rather similar to the main bump of the optimal reflectance in question.

To quantify the similarity of the bumps in width, define the δ*-approximation index* as the absolute value of the difference between the widths of the main bump and the bump of the approximating two-transition optimal reflectance. The similarity of the bumps in positioning can be characterised in terms of a λ_*c*_*-approximation index* defined as the absolute value of the difference between the central wavelengths of the main bump and the bump of the approximating two-transition optimal reflectance. Values for both indices were found to be rather small for many optimal reflectance with three and/or four wavelength transitions.

As an example, consider four-transition optimal reflectances for which the solution (i.e., the two-transition approximating elementary step function) is of the same type. There are 4,392 (77.4%) such reflectances. For 205 (27.4%) of them, both the δ*-*approximation and λ_*c*_*-*approximation indices do not exceed 2 nm. In other words, the main bump of optimal reflectance and the approximating two-transition bump are pretty much the same. At first glance, this corroborates the idea of approximating the optimal reflectances by two-transition elementary step functions. Specifically, for these reflectances, it seems that a slight change in the main transition wavelengths, along with a minor increase in α, is enough to make up for the second bump in terms of yielding the identical *SML* coordinates.

However, this impression is rather misleading. Really, even for this sample, the spread of approximation indices is quite large. Specifically, the average δ*-*approximation index over the 4,392 approximating reflectances was found to be 11.5 nm, with the maximum being 83.2 nm. The maximum λ_*c*_*-*approximation index was 41.5 nm, with the mean being 5.7 nm. One reason for this is that the effect (on the color signal) of shifting the transition wavelength by a fixed amount is very different for different parts of the spectrum. In particular, the effect sharply decreases as one moves towards either end of the spectrum. Therefore, when at least one transition wavelength of the main bump is located near either end of the spectrum, both approximation indices are likely to take on large values.

When the optimal reflectance and the approximating two-transition elementary step function were of different types, the range of the approximation indices was even larger, which further undermines the idea of a two-transition approximation of the optimal reflectances. To put an end to this idea once and for all, let us illustrate its unsolvability with the following example. Consider on the spectrum circle a four-transition elementary step function, *x*(λ; λ_1_, λ_2_, λ_3_, λ_4_), with a wide main bump centred in the middle of the visible spectrum interval (i.e., $\lambda_{c}^{'}=580$ nm) and a narrow secondary bump centred at the glue point of the ends of the visible spectrum interval (i.e., $\lambda_{c}^{''}=380$ or 780 nm) (see Figure 14A). It is clear that for a sufficiently narrow secondary bump the approximating two-transition elementary step function, $x\left(\lambda ;\lambda_{1}^{'},\lambda_{2}^{'}\right)$, will be practically identical to the main bump. Consider now the reflectance, $\tilde{x}\left(\lambda ;\lambda_{1},\lambda_{2},\lambda_{3},\lambda_{4}\right)$, complementary to *x*(λ; λ_1_, λ_2_, λ_3_, λ_4_) (i.e., with the same transition reflectance but of different type) (see Figure 14B). Obviously, its two-transition approximation will be a reflectance complementary to $x\left(\lambda ;\lambda_{1}^{'},\lambda_{2}^{'}\right)$ denote it ($\tilde{x}\left(\lambda ;\lambda_{1}^{'},\lambda_{2}^{'}\right)$). Note, however, that $\tilde{x}\left(\lambda ;\lambda_{1},\lambda_{2},\lambda_{3},\lambda_{4}\right)$ will comprise two identical bumps, the width of which is less than one-half as wide as the width of $\tilde{x}\left(\lambda ;\lambda_{1}^{'},\lambda_{2}^{'}\right)$.

When the bumps are equal, the concept of the main bump loses its meaning. Moreover, whichever one is chosen as the main bump, the approximation indices will be very large. Although we can allow the secondary bump to shift from the glue point in one direction or another, so as to avoid the equality of the bumps of $\tilde{x}\left(\lambda ;\lambda_{1},\lambda_{2},\lambda_{3},\lambda_{4}\right)$, unless this shift is very large, the approximation indices will remain large. Therefore, the two-transition elementary step function $\tilde{x}\left(\lambda ;\lambda_{1}^{'},\lambda_{2}^{'}\right)$ cannot in any way be thought as an approximation of the optimal reflectance $\tilde{x}\left(\lambda ;\lambda_{1},\lambda_{2},\lambda_{3},\lambda_{4}\right)$.

In general, note that the better $x\left(\lambda ;\lambda_{1}^{'},\lambda_{2}^{'}\right)$ approximates *x*(λ; λ_1_, λ_2_, λ_3_, λ_4_), the worse $\tilde{x}\left(\lambda ;\lambda_{1}^{'},\lambda_{2}^{'}\right)$ approximates $\tilde{x}\left(\lambda ;\lambda_{1},\lambda_{2},\lambda_{3},\lambda_{4}\right)$, up to the complete loss of the very meaning of the approximation. In other words, it is impossible in principle to achieve a good two-transition approximation for all three- and four-transition optimal reflectances.

To summarize, although two-transition elementary step functions can be successfully used to approximate the object-color-solid boundary, they should not be considered as even a rough approximation of those optimal reflectances having more than two transition wavelengths.

## Discussion

Being a closed convex set in the color-signal space, the object-color solid is fully determined by its boundary surface, which, in turn, is specified by the optimal reflectances (i.e., those mapping onto this boundary). Although the set of optimal reflectance functions mapping to the boundary has, from the theoretical point of view, been fully characterized (Logvinenko, 2009; Logvinenko & Levin, 2023), the question of how to calculate these functions in a practical way has remained open. The reason for this was, on the one hand, the misconception that such functions are limited to elementary step functions of the second degree, and, on the other hand, the complexity of their calculation. The question of the error that occurs when estimating the object-color solid using such 2-transition step functions has not previously been addressed. We found that, although theoretically significant, this error is rather small when estimated in terms of the distance between the points in the color space.

It would be a mistake, however, to ignore the presence of three- and four-transition elementary step functions among the set of optimal reflectances. Indeed, the difference between the true object-color solid and its two-transition approximation (measured in terms of the radial distance in the color signal space) depends on the choice of units of measurement for the sensors’ outputs. Moreover, the shape of the object-color solid changes with illumination. Any pre-receptor filters, including the atmosphere, will affect the object-color solid. Therefore, it cannot be ruled out a priori that the difference between the true object-color solid and its two-transition approximation (estimated by us only for one particular illumination, assuming an ideally transparent atmosphere, and when choosing very specific units of measurements of the photoreceptors response) for other viewing conditions will have a different quantitative assessment. It is not possible to estimate this difference in general for all viewing conditions. The lack of an invariant specification of this volume in the color signal space (a volume referred to in the vision literature as the object-color solid) leads to the question as to whether or not there is anything fixed and objective about it.

What is objective about the object-color solid is that it represents the classes of metameric reflectances. However, given the variability noted elsewhere in this article, such a representation can hardly be considered consistent. A more satisfactory representation of the classes of metameric reflectances can be created using optimal reflectances (Logvinenko, 2009; Logvinenko & Levin, 2023); because the set of optimal reflectances will remain unchanged regardless of illumination and atmosphere. It will also be independent of the choice of sensor output units. This invariance suggests that the set of optimal reflectances is better suited to be used as the basis of the representation in question than the changeable shape of their image in the color signal space (i.e., the object-color solid). A further benefit is that this set itself has a well-defined geometry.

As shown previously (Logvinenko & Levin, 2023), the set of optimal reflectances can be considered as the boundary of the smooth manifold topologically equivalent to a three-dimensional ball. More specifically, this manifold contains, along with each optimal reflectance *x*_*opt*_, all reflectances of the form α*x*_*opt*_, where α varies from 0 to 1, and only them. The theoretical significance of this ball (of reflectances) follows from the fact that it uniquely represents all the classes of metameric reflectances (thus all the object-colors). In other words, each reflectance is metameric to one of the reflectances comprising this ball. For this reason, it has been termed an object-color atlas (Logvinenko, 2009; Logvinenko & Levin, 2023). Interestingly, mapping this object-color atlas to the color signal space results in the object-color solid. Because such a mapping depends on the viewing conditions, the object-color solid will be different for different viewing conditions. The only thing that unites the object-color solids obtained under different viewing conditions is the object-color atlas whose images they are. Therefore, it seems logical when studying object-colors to turn, not to mutable object-color solids, but rather to the invariant basis hidden behind them—the object-color atlas.

Shifting the focus from the object-color solid to the object-color atlas allows us to address the important issue of how observations by observers with different sensors relate. Consider, for instance, the following simple question. Given two sets of trichromatic sensors, do they produce the same object-color solid or different solids? In particular, how should we distinguish human observers having the same object-color solids from those with different ones? Clearly, the formal overlap of the corresponding volumes in the color signal spaces cannot serve as a criterion because it is not met even for two identical observers unless the same units of measurement are used. In particular, it is unclear what the natural choice of “same” units of measurement might be, for example, for human photoreceptors versus camera sensors. Even if we normalize the sensor responses to the common illumination, the shape of object-color solids (as it was defined elsewhere in this article) will differ. However, if we reformulate this question in terms of the object-color atlas, relating two different sensor sets becomes trivial. Namely, two different sensor sets produce the same object-color atlas when their sets of optimal reflectances are identical. For example, all sensor triplets for which the optimal reflectance sets comprise all and only two-transition reflectances are equivalent in the sense that they have the same object-color atlas. It is noteworthy that the nature of the sensors does not matter (whether they are animal photoreceptors or artificial sensors).

It is possible to introduce spherical coordinates in the object-color atlas by using the αδλ-parameterization of optimal reflectances (Logvinenko, 2009; Logvinenko & Levin, 2023) described in αδλ-Parameterization. In this coordinate system, all the object-color atlases can be represented in a uniform way. As stated, the object-color atlas formed by all, and only two-transition reflectances can be represented as a unit ball. All other object-color atlases can be represented by a volume enclosing a unit ball that extends beyond it along those radii that correspond to the optimal reflectances with more than two transition wavelengths. It is worth noting that such a representation will not depend either on the choice of units of measurement nor on the observation conditions (i.e., illumination, atmosphere). This uniform representation facilitates the comparison of different sensor sets (regardless of their nature). For example, if you want to make artificial vision as close as possible to human vision, it is not necessary to try to make artificial sensors similar to human photoreceptors. Depending on the task facing the designer of artificial vision, it may be enough to ensure that their object-color atlases are identical or at least quite similar.

For observers with equivalent sensor sets (i.e., those which produce identical sets of optimal reflectances) one can establish a one-to-one correspondence between their object-color solids by identifying the color-signal triplets produced by each of the sensor sets in response to the same optimal reflectance. Although this correspondence relates the classes of metameric reflectances of these observers, it does not allow one to predict what color an object will be perceived by one observer based on the knowledge of the color of that object by a second observer. Moreover, we cannot even predict whether the second observer will see two objects as identical in color when these objects are metameric for the first observer (i.e., perceived by her or him as the same color). It is quite possible that objects, metameric for one observer, will appear different in color to another observer (even if these observers have the same object-color atlases). This phenomenon is usually referred to as metamer mismatching (Wyszecki & Stiles, 1982).

One approach to quantitatively assessing metamer mismatching is to evaluate the subset in the object-color atlas of the second observer that comprises all the reflectances which are metameric for the first observer. Alternatively, metamer mismatching can be evaluated in terms of the object-color solid. Specifically, reflectances mapping to a single point of the color-signal space of one observer can map into some subset of points in the color-signal space of another observer. This subset is commonly referred to as the metamer mismatch volume (Wyszecki & Stiles, 1982). Needless to say, because the metamer mismatch volume is defined in color-signal space, it is subject to all the variability discussed above concerning the object-color-solid variability. Hence, the investigation of the metamer mismatch volumes should, generally speaking, be carried out not in the object-color solid, but in the object-color atlas, where their shape does not depend on the observation conditions. This remark is especially relevant if the objective is to compare these volumes in size.

Unfortunately, there is, as yet, no method for determining the exact metamer mismatch volumes even in the object-color solid. The reason for this lack is of the same nature as in the case of object-color solid. The fact is that from the formal point of view, metamer-mismatching volumes are three-dimensional cross-sections of a sort of six-dimensional object-color solid (Logvinenko & Levin, 2023). The doubling of dimension is due to the fact that the sensors of both observers are combined. It follows that, in its essence, estimating metamer mismatch volumes will be reduced to estimating a six-dimensional object-color solid. Because we have some computational problems evaluating the object-color solid in the three-dimensional space, is it any wonder that we are not able to do this easily in six-dimensional space? We believe that the approach presented in this article could be developed into an algorithm for calculating exact metamer mismatch volumes.

As in the case of object-color solid, however, the computation becomes significantly easier if one restricts oneself to an approximate estimation. Specifically, one can begin with a five-transition approximation of metamer mismatch volumes (an analog of the two-transition approximation of the object-color solid). Calculations (based on the five-transition approximation) (Logvinenko, Funt, & Godau, 2012; Logvinenko, Funt, & Godau, 2014) showed that the metamer mismatching volumes are much larger than one would expect based only on common sense and intuition. Such five-transition approximations of metamer-mismatch volumes have been shown to provide important information that can be used in color imaging (Roshan & Funt, 2020; Roshan & Funt, 2022), color rendering (Mirzaei & Funt, 2015b), and other color science applications (Funt & Roshan, 2021).

Unfortunately, the accuracy of these five-transition approximations relative to the six-transition metamer-mismatch volumes remains unknown. Solving the five-dimensional analog of Equation 9 yields, as a rule, reflectances with more than five transitions. Therefore, the true metamer mismatch volumes can be expected to be even larger. Establishing a practical algorithm for determining the true metamer-mismatch volumes will facilitate determining how accurate the five-transition approximation actually is. However, this is a topic for future research that will involve developing a method for solving an inverse problem similar to the one described above for solving the Schrödinger problem.

In summary, while there have been various approximations made of the object-color solid, the full extent of the object-color solid was previously unknown. The theory, algorithm and results described here establish, for the first time, the true object-color solid. The theory and algorithm also point the way to establishing a method for computing true metamer mismatch volumes. Thus the theory and methods presented in this article not only establish the true shape of the object-color solid, but their significance extends to other areas of color science and technology.

## Figures and Tables

**Figure A2.**
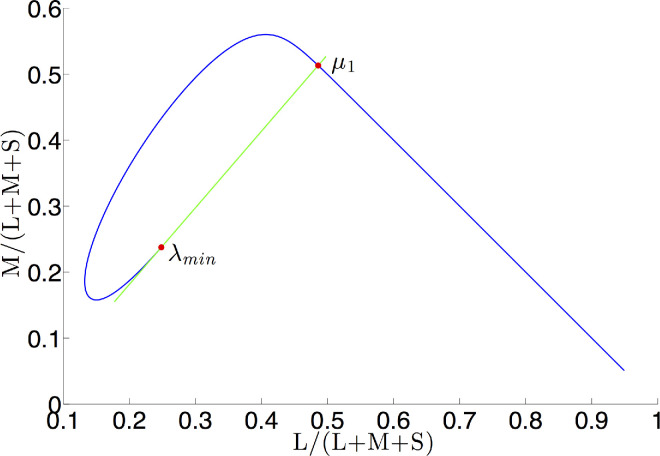
Tangent line through c→(λ min ) crosses the spectral locus at $\vec{c}\left(\mu_{1}\right)$.

**Figure A3.**
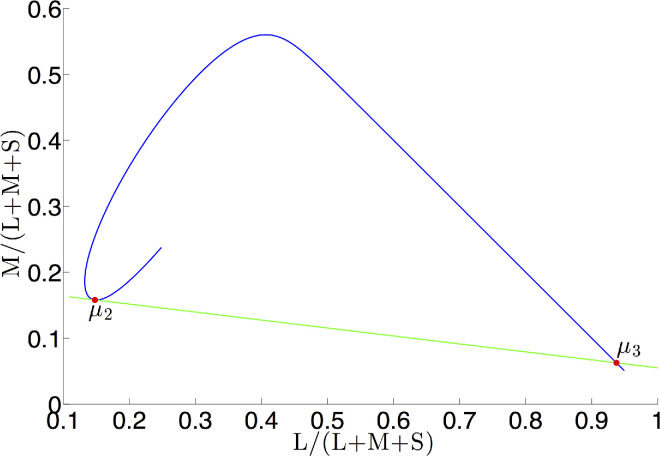
Tangent line at μ_2_ crossing the spectral locus at c→(λ max ) and $\vec{c}\left(\mu_{3}\right)$. Recall that the spectral locus has two hooks, although the one at the long-wave end is not visible in the plot.

**Figure A4.**
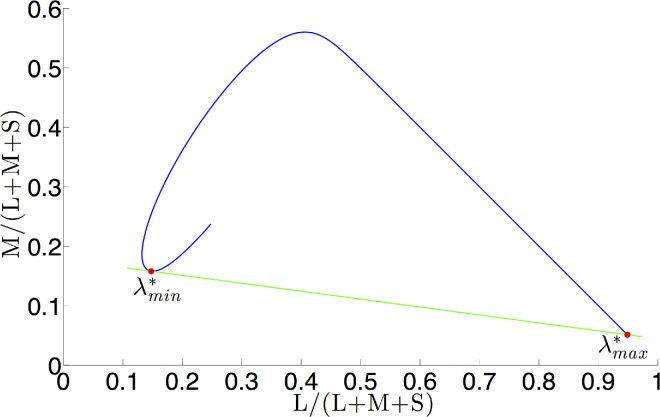
Tangent line to the spectral locus at c→(λ min *) and c→(λ max *). This tangent line defines the ends of the effective visible spectrum.

**Figure A5.**
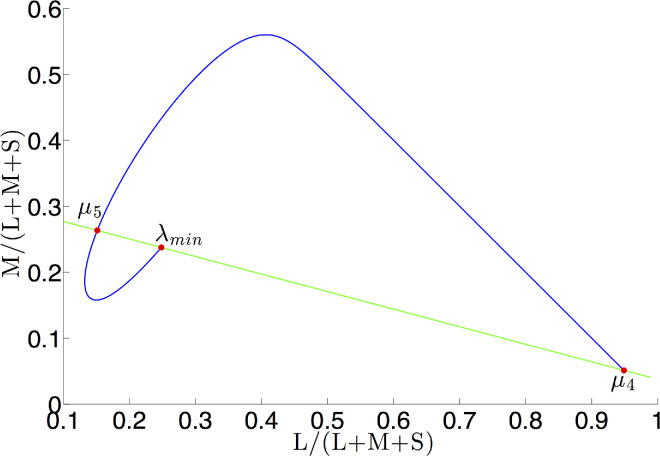
The line through c→(λ min ) and tangent at $\vec{c}\left(\mu_{4}\right)$ crosses the spectral locus at $\vec{c}\left(\mu_{5}\right)$ as well.

## Appendix A: Appendix A: Parameterizing optimal reflectances with two transition wavelengths

To parameterize the optimal reflectances in terms of two transition wavelengths, the crucial step is to establish the subset of ordered wavelength pairs Λ^2^ = {(λ_1_, λ_2_): λ_min _ ⩽ λ_1_ ⩽ λ_2_ ⩽ λ_max _}, for which there is a one-to-one map $\Lambda^{2}\to O$, where O is the set of optimal reflectances of type T1. By symmetry, we only need to consider optimal reflectances of one type. As pointed out in the main text, every pair (λ_1_, λ_2_) in Λ^2^ induces an optimal reflectance that is determined by the intersections of the plane passing through the two points, $\vec{C}\left(\lambda_{1}\right)$ and $\vec{C}\left(\lambda_{2}\right)$, on the spectral curve. This gives us a map (denote it r, and denote the number of transitions of the optimal reflectance function r(λ_1_, λ_2_)) as *N*(λ_1_, λ_2_)) of Λ^2^ onto O; however, the difficulty is that r is not bijective (i.e., not one-to-one).

Figure 4 shows that some pairs from Λ^2^ map to optimal reflectances having more than two transition wavelengths. As mentioned, if, for example, a pair (λ_1_, λ_2_) induces an optimal reflectance, *x*_*opt*_, with four transition wavelengths λ_1_ ⩽ λ_2_ ⩽ λ_3_ ⩽ λ_4_ then all other ordered pairings of these four wavelengths (i.e., (λ_3_, λ_4_), (λ_1_, λ_3_) and so on) will also induce the same *x*_*opt*_. Therefore, all these pairs (i.e., taken from (λ_1_, λ_2_, λ_3_, λ_4_)) map to *x*_*opt*_, thereby violating the bijectivity of r.

Fortunately, it is possible to delimit a region within Λ^2^ for which the map r becomes bijective. In Figure A1, the area overlaid with dots is what we show below should be excluded from Λ^2^ to make map r bijective. Denote the excluded area *E* and let us call $\bar{\Lambda^{2}}=$Λ^2^∖*E* the *admissible region* of Λ^2^. Our goal here is to determine the set of (λ_1_, λ_2_) that form this region.

Given any (λ_1_, λ_2_) in $\bar{\Lambda^{2}}$ (λ_1_ ≠ λ_2_), the points $\vec{c}\left(\lambda_{1}\right)$ and $\vec{c}\left(\lambda_{2}\right)$ on the spectral locus in chromaticity space determine a line in the chromaticity plane that may intersect the spectral locus at one or two additional points: say, $\vec{c}\left(\lambda_{3}\right)$ and possibly $\vec{c}\left(\lambda_{4}\right)$, with λ_1_ < λ_2_ < λ_3_ < λ_4_. In either case, we define r to map the pair (λ_1_, λ_2_) to the corresponding optimal reflectance with three (respectively, four) transition wavelengths, λ_1_, λ_2_, and λ_3_ or λ_1_, λ_2_, λ_3_, and λ_4_.

For the sake of generality one can assume that when λ_1_ = λ_2_ (i.e., when (λ_1_, λ_2_) belongs to the diagonal Δ = {(λ, λ): λ ∈ [λ_min _, λ_max _]} of $\bar{\Lambda^{2}}$) then $r\left(\lambda ,\lambda \right)$ is the optimal reflectance induced by the tangent line to the spectral locus at λ. Indeed, as wavelength λ_2_ approaches wavelength λ_1_, the secant line to the spectral locus defined by the points $\vec{c}\left(\lambda_{1}\right)$ and $\vec{c}\left(\lambda_{2}\right)$ becomes the tangent at $\vec{c}\left(\lambda_{1}\right)$. Similarly, λ_3_ = λ_4_ denotes a tangency at $\vec{c}\left(\lambda_{3}\right)$.

If the tangent to the spectral locus at $\vec{c}\left(\lambda \right)$ intersects the spectral locus at points $\vec{c}\left(\lambda_{2}\right)$ and perhaps $\vec{c}\left(\lambda_{3}\right)$ then it seems natural to have r map (λ, λ) to an optimal reflectance with transition wavelengths λ_2_, and possibly λ_3_. However, if the tangent line at $\vec{c}\left(\lambda \right)$ does not intersect the spectral locus at any other point, then $r\left(\lambda ,\lambda \right)$ is the perfect reflector (or, the perfect absorber). If the tangent line at $\vec{c}\left(\lambda \right)$ intersects the spectral locus at only one other point, say, $\vec{c}\left(\lambda_{2}\right)$, then $r\left(\lambda ,\lambda \right)$ is *x*_1_(λ; λ_2_). If it intersects at two points $\vec{c}\left(\lambda_{2}\right)$ and $\vec{c}\left(\lambda_{3}\right)$, then $r\left(\lambda ,\lambda \right)=x_{1}\left(\lambda ;\lambda_{2},\lambda_{3}\right)$.

It should be noted that, for any line intersecting the spectral locus at exactly one point, there is a tangent to the spectral locus that determines the same optimal reflectance function. Given a line through a point $\vec{c}\left(\lambda_{0}\right)$ that has no other point in common with the spectral locus, rotating this line clockwise or counterclockwise one can make the line touch the spectral locus at exactly one point. Hence, the map r is surjective since for any optimal reflectance *x*_*opt*_ there is a pair (λ_1_, λ_2_) (possibly with equal λ*s*) such that $r\left(\lambda_{1},\lambda_{2}\right)=x_{opt}$.

Consider now in more detail the r-image of the diagonal Δ, i.e., $r\left(\Delta \right)$. To begin, the wavelength λ where a tangent to the spectral locus at $\vec{c}\left(\lambda_{t}\right)$ intersects the spectral locus can be found by solving the following equation with respect to λ:

(34) $$T\left(\lambda ;\lambda_{t}\right)\equiv \frac{l^{'}\left(\lambda_{t}\right)}{m^{'}\left(\lambda_{t}\right)}-\frac{l\left(\lambda \right)-l\left(\lambda_{t}\right)}{m\left(\lambda \right)-m\left(\lambda_{t}\right)}=0,$$

where *m*(λ) and *l*(λ) are the components of vector $\vec{c}\left(\lambda \right)$ (i.e., $\vec{c}\left(\lambda \right)=\left(m(\lambda ),l(\lambda )\right)$) and *m*′ and *l*′ are the corresponding derivatives.

### Admissible region along the diagonal

To determine the number of transition wavelengths along the diagonal, Δ, we vary λ_*t*_ from λ_min_ to λ_max_ and solve for the intersections of the tangent line at λ_*t*_ with the spectral locus using (Equation 34). Beginning with λ_*t*_ = λ_min _, direct calculation shows that the tangent to the spectral locus intersects the locus at only one other wavelength λ = μ_1_ = 538.2160 nm (i.e., *T*(μ_1_; λ_min _) = 0). See Figure A2. This means there is a single wavelength transition, so *N*(λ_min_, λ_min_) = 1. As λ_*t*_ increases beyond λ_min_, the number of transitions remains unchanged until λ_*t*_ reaches μ_2_ = 418.5598 nm (see Figure A3), at which point the tangent line intersects the curve at μ_3_ = 664.9525 nm and $\vec{c}\left(\lambda_{\text{max}}\right)$. Note that, as discussed in the context of Figure 2, the spectral locus bends inward in the long-wave range, although the scale is not fine enough for the bend to be seen in Figure A3. Therefore, lines that are tangent to the spectral locus in the interval [λ_min_, μ_2_) cross the curve at only one location in the interval [μ_1_, μ_3_).

Starting from μ_2_, the tangent lines start to cross the spectral locus at two locations. This continues until λ_*t*_ reaches $\lambda_{\min}^{*}$ = 418.6862 nm, at which point the line also becomes tangent in the long wavelength end of the curve at $\lambda_{\max}^{*}$ = 700.8941 nm (Figure A4). The values for λ min * and λ max * are found by using (Equation 34) twice to obtain and solve the two equations describing a line that is tangent to the spectral locus at both c→(λ min *) and c→(λ max *): T(λ min *;λ max *)=0 and T(λ max *;λ min *)=0. Thus, for $\lambda_{t}\in \left[\mu_{2},\lambda_{\min}^{*}\right)$ tangents to the spectral locus cross the curve twice at ${\hat{\lambda }}_{1}\in \left(\lambda_{\max}^{*},\lambda_{\max}\right]$ and ${\hat{\lambda }}_{2}\in \left[\mu_{3},\lambda_{\max}^{*}\right)$, which can be solved for using (Equation 34). This section of the diagonal is excluded from the admissible region as it can be uniquely represented by coordinate pairs $\left({\hat{\lambda }}_{1},{\hat{\lambda }}_{2}\right)$.

The portion of the spectral locus between $\vec{c}\left(\lambda_{\min}^{*}\right)$ and $\vec{c}\left(\lambda_{\max}^{*}\right)$ is such that the tangent line at any point along it does not have any other point in common with the spectral locus, since for λt∈[λ min *,λ max *] (Equation 34) has no solution. Hence, in Figure A1 the points (λ_*t*_, λ_*t*_) along the diagonal for this interval correspond to zero-transition optimal reflectance functions and are indicated in purple. Because zero-transition reflectances are all represented by the single point $\left(\lambda_{\min}^{*},\lambda_{\min}^{*}\right)$, the remaining diagonal points $\left\{\left(\lambda_{t},\lambda_{t}\right)\;|\;\lambda_{t}\in \left(\lambda_{\min}^{*},\lambda_{\max}^{*}\right)\right\}$ are excluded from the admissible region.

The portion of the spectral locus between $\vec{c}\left(\lambda_{\min}^{*}\right)$ and $\vec{c}\left(\lambda_{\max}^{*}\right)$ combined with the interval connecting its ends (i.e., $\vec{c}\left(\lambda_{\min}^{*}\right)$ and $\vec{c}\left(\lambda_{\max}^{*}\right)$) makes a closed contour that outlines the *chromaticity gamut*, that is, the set of the chromaticities of all possible lights. The interval $\left[\lambda_{\min}^{*},\lambda_{\max}^{*}\right]$ has been called the effective visible spectrum interval (Logvinenko, 2013).

As λ_*t*_ moves beyond $\lambda_{\max}^{*}$, two new intersections emerge in the short wavelength end of the spectrum. This continues to happen until λ_*t*_ reaches μ_4_ = 700.8944 nm (see Figure A5), at which point the tangent line crosses the spectral locus at λ_min _ and μ_5_ = 452.1641. Thus, for $\lambda_{t}\in \left(\lambda_{\max}^{*},\mu_{4}\right]$ tangential lines cross the curve at two locations, $\lambda_{1}\in \left(\lambda_{\min}^{*},\mu_{5}\right]$ and $\lambda_{2}\in \left[\lambda_{\min}^{*},\lambda_{\min}\right)$, that can be solved using (Equation 34). Because these tangents can be uniquely represented by off-diagonal points with coordinates (λ_1_, λ_2_), the fragment of the diagonal in the interval $(\lambda_{\max}^{*}$, μ_4_] is not included in the admissible region shown in Figure A1.

As λ_*t*_ moves past μ_4_, the tangent line crosses the spectral locus at only one location. This continues until the tangent line passes through μ_6_ = 564.9482 and λ_max _ at λ_*t*_ = μ_7_ = 714.6564. Solving Equation 34 for λ_*t*_ ∈ (μ_4_, μ_7_), we obtain the location of the crossing in the interval (μ_5_, μ_6_). For λ_*t*_ past μ_7_, the tangent lines cross the spectral locus at two locations, and by the same reasoning as above, the fragment of the diagonal in the interval [μ_7_, λ_max _] is not included in the admissible region. Table A1 summarizes the numerical values of the parameters described above. The first three rows of Table A2 give the bounds of the intervals of the admissible region on the diagonal.

**Table A1.** Numerical values in nanometers of the parameters used to describe the admissible region. Note that μ_4_ = 700.8944 nm and $\lambda_{\max}^{*}=700.8941$ nm only differ at the fourth decimal place.

	
$\lambda_{\min}^{*}$	418.6862
μ_1_	538.2160
μ_2_	418.5598
μ_3_	664.9525
μ_4_	700.8944
μ_5_	452.1641
μ_6_	564.9482
μ_7_	714.6564
$\lambda_{max}^{*}$	700.8941

**Table A2.** The admissible region (shown as the nondotted area in Figure A1) is characterized by the set of points {(λ_1_, λ_2_)}, where λ_1_ and λ_2_ are specified according to this table. For a λ_1_ in an interval from the left column, λ_2_ is restricted to lie within the corresponding interval in the second column. The first three rows where λ_2_ = λ_1_, describe the segments of the diagonal belonging to the admissible region. The remaining rows describe the off-diagonal portions of the admissible region. For example, the fourth row indicates that for λ_1_ in the interval (380, 452.1641), λ_2_ must be from the interval (λ_1_, λ_*w*_(380; λ_1_)) in order for the coordinate pair (λ_1_, λ_2_) to belong to the admissible region.

Transition 1 (λ_1_) wavelength interval (nm)	Transition 2 (λ_2_) wavelength interval
λ_min_ = 380.0000 < λ_1_ < 418.5598 = μ_2_	λ_1_
λ min *=418.6862=λ1	λ_1_
μ_4_ = 700.8944 < λ_1_ < 714.6564 = μ_7_	λ_1_
λ_min_ = 380.0000 < λ_1_ < 452.1641 = μ_5_	λ_1_ < λ_2_ < λ_*w*_(λ_min _; λ_1_)
μ_5_ = 452.1641 < λ_1_ < 564.9482 = μ_6_	λ_1_ < λ_2_ < λ_*t*_(λ_1_)
μ_6_ = 564.9482 < λ_1_ < 664.9525 = μ_3_	λ_1_ < λ_2_ < λ_max _
μ3=664.9525<λ1<700.8941=λ max *	λ_1_ < λ_2_ < λ_*t*_(λ_1_)

### Admissible region off the diagonal

Having established the intervals on the diagonal of the λ_1_λ_2_-plane that belong to the admissible region $\bar{\Lambda^{2}}$, what remains is to determine the off-diagonal points that belong to it as well. To do so, we consider λ_1_ cross-sections of the λ_1_λ_2_–plane (i.e., fix λ_1_ and vary λ_2_), and describe the valid λ_2_ interval for each given value of λ_1_.

To begin, consider a line that crosses the spectral locus at $\vec{c}\left(\lambda_{1}\right)$ and $\vec{c}\left(\lambda_{2}\right)$, and let λ be the longest wavelength at which this line crosses the locus. This wavelength is determined as the largest root of the following equation:

(35) $$\begin{array}{c} \;W\left(\lambda ;\lambda_{1},\lambda_{2}\right)=\frac{m\left(\lambda \right)-m\left(\lambda_{2}\right)}{m\left(\lambda_{1}\right)-m\left(\lambda_{2}\right)}-\frac{l\left(\lambda \right)-l\left(\lambda_{2}\right)}{l\left(\lambda_{1}\right)-l\left(\lambda_{2}\right)}=0. \end{array}$$

For λ_1_ ∈ [λ_min_, μ_5_) the admissible interval for λ_2_ is given by (λ_1_, λ_*w*_), where λ_*w*_(λ_min _, λ_1_) is the largest root of *W*(λ; λ_min _, λ_1_).

To understand why this is so, first recall that μ_5_ = 452.1641 nm is the wavelength at which the line through *c*(λ_min_) and tangent to the spectral locus at λ = μ_4_ crosses the spectral locus (see Figure A5). Consider now some λ′ ⩾ λ_*w*_(λ_min _, λ_1_). The line through $\vec{c}\left(\lambda_{1}\right)$ and $\vec{c}\left(\lambda^{'}\right)$ in Figure A5 intersects the spectral locus at a third point $\vec{c}\left(\lambda {}^{*}\right)$ such that λ_min _ < λ* < λ_1_. However, the pair (λ*, λ_1_) will already have appeared under such a procedure for determining pairs (namely, in the case when the value λ* was taken as λ_1_, and λ′ as λ_2_). Thus, to preserve the bijectivity of $\bar{\Lambda^{2}}$, we specify only the following subset as belonging to the admissible region:

(36) $$\begin{array}{ccc} & & \{\left(\lambda_{1},\lambda_{2}\right)\;|\;\lambda_{1}\in \left[\lambda_{\min},\mu_{5}\right)\;\text{and}\\ & & \;\lambda_{2}\in \left(\lambda_{1},\lambda_{w}\left(\lambda_{\min},\lambda_{1}\right)\right)\}\in \bar{\Lambda^{2}}\; \end{array}$$

For λ_1_ ∈ [μ_5_, μ_6_), we have λ_2_ ∈ (λ_1_, λ_*t*_), where λ_*t*_ > λ_1_ is the wavelength at which the line through λ_1_ becomes tangent to the spectral locus, with λ_*t*_ being determined by (Equation 34). The points in this section of the admissible region are:

(37) $$\begin{array}{c} \;\left\{\left(\lambda_{1},\lambda_{2}\right)\;|\;\lambda_{1}\in \left[\mu_{5},\mu_{6}\right)\;\text{and}\;\lambda_{2}\in \left(\lambda_{1},\lambda_{t}\left(\lambda_{1}\right)\right)\right\}\in \bar{\Lambda^{2}}\; \end{array}$$

As before, λ2∉[λt,λ max ] since for these values the line through $\vec{c}\left(\lambda_{1}\right)$ and $\vec{c}\left(\lambda_{2}\right)$ would cross the spectral locus at λ ⩽ λ_2_, resulting in coordinate pairs (λ_1_, λ_2_) that have already been allocated.

When λ_1_ ∈ [μ_6_, μ_3_), the cross-section of the admissible region is given by λ_2_ ∈ (λ_1_, λ_max_]. Recall (see Figure A3) that μ_3_ = 664.9525 nm is the wavelength at which the line through λ_max_ and tangent in the short wavelength region (i.e., at μ_2_) crosses the spectral locus. Unlike the previous cases, we do not need to exclude points from the interval (λ_1_, λ_max_] because the line through $\vec{c}\left(\lambda_{1}\right)$ and $\vec{c}\left(\lambda_{2}\right)$ does not cross the spectral locus at any other location. Thus, this section of the admissible region is:

(38) $$\;\left\{\left(\lambda_{1},\lambda_{2}\right)\;|\;\lambda_{1}\in \left[\mu_{6},\mu_{3}\right)\;\text{and}\;\lambda_{2}\in \left(\lambda_{1},\lambda_{\max}\right]\right\}\in \bar{\Lambda^{2}}$$

Finally, if λ1∈[μ3,λ max *), then $\lambda_{2}\in \left(\lambda_{1},\lambda_{1}^{*}\right)$, where $\lambda_{1}^{*}>\lambda_{1}$ is the second intersection of a line that goes through λ_1_ and is tangent to the spectral locus at the short wavelength end. To find $\lambda_{1}^{*}$, we first solve for λ_*t*_(λ_1_) using (Equation 34). Then, with λ_*t*_ known, we solve the same equation for the largest wavelength (i.e., solve $T(\lambda_{1}^{*};\lambda_{t})=0$). Note that for λ_2_ > λ_*t*_(λ_1_) the line through $\vec{c}\left(\lambda_{1}\right)$ and $\vec{c}\left(\lambda_{2}\right)$ crosses the spectral locus at λ < λ_1_, resulting in coordinate pairs that have already been allocated. For this reason, the interval for λ_2_ does not include (λ_*t*_(λ_1_), λ_max_]. This section of the admissible region is then:

(39) $$\begin{array}{c} \;\left\{\left(\lambda_{1},\lambda_{2}\right)\;|\;\lambda_{1}\in \left[\mu_{3},\lambda_{\max}^{*}\right)\;\text{and}\;\lambda_{2}\in \left(\lambda_{1},\lambda_{t}\left(\lambda_{1}\right)\right)\right\}\in \bar{\Lambda^{2}} \end{array}$$

For λ1∈[λ max *,λ max ) and λ_2_ > λ_1_, the crossings of the line though $\vec{c}\left(\lambda_{1}\right)$ and $\vec{c}\left(\lambda_{2}\right)$ are all less than λ_1_. Thus, there are no points (λ_1_, λ_2_) with λ_2_ > λ_1_, that belong to the admissible region for λ1≥λ max *.

To summarize, the admissible region can be divided into several sections where each section can be described by a set of points (λ_1_, λ_2_) as defined in Table A2.

## Appendix B: Appendix B: Solving the inverse problem

In Identifying the object-color solid via solving Schrödinger’s problem, the inverse problem was formulated as follows. Given a direction (i.e., a ray from the origin) in color signal space, find the optimal reflectance that maps to some point along that ray. In other words, given a color signal ${\bar{z}}^{*}=\left(z_{1}^{*},z_{2}^{*},z_{3}^{*}\right)$, the problem is to find the optimal reflectance *x*_*opt*_(λ) such that $\Phi \left(x_{opt}\right)=\kappa \left(z_{1}^{*},z_{2}^{*},z_{3}^{*}\right)$, where κ is some unknown constant (see Equation 33). More specifically, it is required to solve the following equations for the unknowns λ_1_, λ_2_, and κ:

(40) $$\begin{array}{c} \;\int_{\lambda_{\min}}^{\lambda_{\max}}x\left(\lambda ;\lambda_{1},\lambda_{2}\right)I\left(\lambda \right)s_{i}\left(\lambda \right)d\lambda =\kappa z_{i}^{*}\;\left(i=1,2,3\right),\; \end{array}$$

where *x*(λ; λ_1_, λ_2_) is an optimal reflectance specified by a (λ_1_, λ_2_) belonging to the admissible region as described in Appendix A. Naturally, we will use numerical methods to find an approximate solution instead of the exact one, the existence of which was proved in Appendix A.

Let us rewrite Equation 40 as

(41) $$\Phi \left(\bar{\lambda }\right)=\kappa {\bar{z}}^{*},$$

where $\bar{\lambda }=\left(\lambda_{1},\lambda_{2}\right)$ and ${\bar{z}}^{*}=\left(z_{1}^{*},z_{2}^{*},z_{3}^{*}\right)$. Here Φ is, actually, the same as the color signal map, which becomes a map **R**^2^ → **R**^3^ after parameterizing the optimal reflectances with transition wavelengths λ_1_ and λ_2_. It is convenient to change from Cartesian coordinates *z*_1_, *z*_2_, and *z*_3_ to the spherical coordinates ρ, α_1_, and α_2_, having radius ρ, and inclination (α_1_) and azimuth (α_2_) angles defined as

(42) $$\begin{array}{ccc} \rho & = & \sqrt{z_{1}^{2}+z_{2}^{2}+z_{3}^{2}},\\ \alpha_{1} & = & \arccos\left(\frac{z_{3}}{\sqrt{z_{1}^{2}+z_{2}^{2}+z_{3}^{2}}}\right),\\ \alpha_{2} & = & sgn\left(z_{2}\right)\arccos\left(\frac{z_{1}}{\sqrt{z_{1}^{2}+z_{2}^{2}}}\right).\; \end{array}$$

The range of the inclination and azimuth will be restricted to the following intervals: 0 ⩽ α_1_ ⩽ π and −π < α_2_ ⩽ π.

In these spherical coordinates, Equation 41 leads to the following

(43) $$F\left(\bar{\lambda }\right)={\bar{\alpha }}^{*},$$

where ${\bar{\alpha }}^{*}=\left(\alpha_{1}^{*},\alpha_{2}^{*}\right)$ is the direction corresponding to the point ${\bar{z}}^{*}$.

Here the two-variable function *F* = *T*○Φ: **R**^2^ → **R**^2^ is a composition of Φ and part of the coordinate transformation (Equation 42) involving α_1_ and α_2_ (specifically, *T* stands for the last two lines in Equation 42). Relating $\bar{\alpha }=\left(\alpha_{1},\alpha_{2}\right)$ and $\bar{\lambda }=\left(\lambda_{1},\lambda_{2}\right)$, function *F* is invertible in a neighborhood, $U\left(\bar{\lambda }{}^{0}\right)$, of a point, $\bar{\lambda }{}^{0}$, provided that the Jacobian matrix, **J**_*F*_, of *F* at $\bar{\lambda }{}^{0}$ is invertible, i.e., $det\left(J_{F}\left(\bar{\lambda }{}^{0}\right)\right)\neq 0$ .

Suppose ${\bar{\alpha }}^{*}\in F\left(U\left(\bar{\lambda }{}^{0}\right)\right)$, $F\left(\bar{\lambda }{}^{*}\right)={\bar{\alpha }}^{*}$, $F\left(\bar{\lambda }{}^{0}\right)=$ ${\bar{\alpha }}^{0}$, and $\Delta \bar{\lambda }={\bar{\lambda }}^{*}-\bar{\lambda }{}^{0}$, then

$$\begin{array}{c} F\left(\bar{\lambda }{}^{*}\right)=F\left(\bar{\lambda }{}^{0}+\Delta \bar{\lambda }\right)\approx F\left(\bar{\lambda }{}^{0}\right)+J_{F}\left(\bar{\lambda }{}^{0}\right)\Delta \bar{\lambda }, \end{array}$$

or

$$\begin{array}{c} {\bar{\lambda }}^{*}-\bar{\lambda }{}^{0}\approx J_{F}^{-1}\left(\bar{\lambda }{}^{0}\right)\left(\bar{\alpha }{}^{*}-\bar{\alpha }{}^{0}\right), \end{array}$$

where $J_{F}^{-1}\left(\bar{\lambda }{}^{0}\right)$ is the matrix inverse of the Jacobian matrix $J_{F}\left(\bar{\lambda }{}^{0}\right)$ of *F* at $\bar{\lambda }{}_{0}$.

As a result, we have an approximate formula for the solution to Equation 43:

(44) $$\begin{array}{c} {\bar{\lambda }}^{*}\approx {\bar{\lambda }}^{1}=\bar{\lambda }{}^{0}+J_{F}^{-1}\left(\bar{\lambda }{}^{0}\right)\left({\bar{\alpha }}^{*}-{\bar{\alpha }}^{0}\right).\; \end{array}$$

The smaller $\Delta \bar{\lambda }$, the better the accuracy of this approximation.

As we do not know ${\bar{\lambda }}^{*}$ (thus, $\Delta \bar{\lambda }$), we start from a $\bar{\lambda }{}^{0}$ such that the corresponding direction ${\bar{\alpha }}^{0}$ is close to ${\bar{\alpha }}^{*}$ so $\left({\bar{\alpha }}^{*}-{\bar{\alpha }}^{0}\right)$ is small, and then iterate using ${\bar{\lambda }}^{1}$ as the new initial value. In particular, we have the following iteration formula for a root of (Equation 43)

(45) $$\begin{array}{c} {\bar{\lambda }}^{*}\approx {\bar{\lambda }}^{n+1}={\bar{\lambda }}^{n}+J_{F}^{-1}\left(\bar{\lambda }{}^{n}\right)\left({\bar{\alpha }}^{*}-{\bar{\alpha }}^{n}\right).\; \end{array}$$

A sufficiently accurate approximation to ${\bar{\lambda }}^{*}$ is reached in a few iterations. This procedure is simply an application of Newton’s method for solving nonlinear equations.

Applying the chain rule, Jacobian $J_{F}\left(\bar{\lambda }\right)$ can be expressed as

(46) $$\begin{array}{c} \;J_{F}\left(\bar{\lambda }\right)=J_{T\circ \Phi }\left(\bar{\lambda }\right)=J_{T}\left(\Phi \left(\bar{\lambda }\right)\right)J_{\Phi }\left(\bar{\lambda }\right).\; \end{array}$$

From (Equation 42) we get by direct differentiation:

(47) $$\begin{array}{ccc} J_{T}\left(\Phi \left(\bar{\lambda }\right)\right) & \;= & \left\{\begin{array}{ccc} \frac{\partial \alpha_{1}}{\partial z_{1}} & \frac{\partial \alpha_{1}}{\partial z_{2}} & \frac{\partial \alpha_{1}}{\partial z_{3}}\\ \frac{\partial \alpha_{2}}{\partial z_{1}} & \frac{\partial \alpha_{2}}{\partial z_{2}} & \frac{\partial \alpha_{2}}{\partial z_{3}} \end{array}\right\}\\ & \;= & \left\{\begin{array}{ccc} \frac{z_{1}z_{3}}{\rho^{2}\sqrt{z_{1}^{2}+z_{2}^{2}}} & \frac{z_{2}z_{3}}{\rho^{2}\sqrt{z_{1}^{2}+z_{2}^{2}}} & -\frac{\sqrt{z_{1}^{2}+z_{2}^{2}}}{\rho^{2}}\\ \frac{-z_{2}}{z_{1}^{2}+z_{2}^{2}} & \frac{z_{1}}{z_{1}^{2}+z_{2}^{2}} & 0 \end{array}\right\},\; \end{array}$$

where $\rho^{2}=z_{1}^{2}+z_{2}^{2}+z_{3}^{2}$.

The entries of the Jacobian matrix $J_{\Phi }\left(\bar{\lambda }\right)$ can be found by differentiating the following equation:

(48) $$z_{i}=z_{i}\left(\lambda_{1},\lambda_{2}\right)=\int_{\lambda_{1}}^{\lambda_{2}}s_{i}\left(\lambda \right)d\lambda +\int_{\lambda_{3}}^{\lambda_{4}}s_{i}\left(\lambda \right)d\lambda .$$

Specifically, the partial derivative of *z*_*i*_ with respect to λ_1_ is

(49) $$\frac{\partial z_{i}}{\partial \lambda_{1}}=-s_{i}\left(\lambda_{1}\right)+s_{i}\left(\lambda_{4}\right)\frac{\partial \lambda_{4}}{\partial \lambda_{1}}-s_{i}\left(\lambda_{3}\right)\frac{\partial \lambda_{3}}{\partial \lambda_{1}}.$$

Likewise, the partial derivative of *z*_*i*_ with respect to λ_2_ is given as

(50) $$\frac{\partial z_{i}}{\partial \lambda_{2}}=s_{i}\left(\lambda_{2}\right)+s_{i}\left(\lambda_{4}\right)\frac{\partial \lambda_{4}}{\partial \lambda_{2}}-s_{i}\left(\lambda_{3}\right)\frac{\partial \lambda_{3}}{\partial \lambda_{2}}.$$

Recall that the third and fourth transition wavelengths λ_3_ and λ_4_ are functions of λ_1_ and λ_2_. In fact, λ_3_ and λ_4_ are the roots of the function *g*(λ) in Equation 9, the coefficients, *k*_*i*_, of which are determined by λ_1_ and λ_2_ via Equation 24. Thus, we have

(51) $$\begin{array}{ccc} \frac{\partial \lambda_{4}}{\partial \lambda_{1}} & = & \sum_{i=1}^{i=3}\frac{\partial \lambda_{4}}{\partial k_{i}}\frac{\partial k_{i}}{\partial \lambda_{1}};\;\;\;\frac{\partial \lambda_{4}}{\partial \lambda_{2}}=\sum_{i=1}^{i=3}\frac{\partial \lambda_{3}}{\partial k_{i}}\frac{\partial k_{i}}{\partial \lambda_{2}};\\ \frac{\partial \lambda_{3}}{\partial \lambda_{1}} & = & \sum_{i=1}^{i=3}\frac{\partial \lambda_{3}}{\partial k_{i}}\frac{\partial k_{i}}{\partial \lambda_{1}};\;\;\;\frac{\partial \lambda_{3}}{\partial \lambda_{2}}=\sum_{i=1}^{i=3}\frac{\partial \lambda_{3}}{\partial k_{i}}\frac{\partial k_{i}}{\partial \lambda_{2}}.\; \end{array}$$

The partial derivatives of λ_3_ and λ_4_ with respect to *k*_*i*_ were evaluated in Equation 26. So one gets

(52) $$\begin{array}{c} \;\frac{\partial \lambda_{j}}{\partial k_{i}}=-\frac{s_{i}\left(\lambda_{j}\right)}{k_{1}s_{1}^{'}\left(\lambda_{j}\right)+k_{2}s_{2}^{'}\left(\lambda_{j}\right)+k_{3}s_{3}^{'}\left(\lambda_{j}\right)},\;j=3,4. \end{array}$$

The coefficients *k*_*i*_ are given in Equation 24 as functions of λ_1_ and λ_2_. Differentiating Equation 24 yields

(53) $$\begin{array}{ccc} & & \frac{\partial k_{i}}{\partial \lambda_{1}}={\left(-1\right)}^{i-1}\left|\begin{array}{cc} s_{p}^{'}\left(\lambda_{1}\right) & s_{q}^{'}\left(\lambda_{1}\right)\\ s_{p}\left(\lambda_{2}\right) & s_{q}\left(\lambda_{2}\right) \end{array}\right|\;\text{and}\\ & & \frac{\partial k_{i}}{\partial \lambda_{2}}={\left(-1\right)}^{i-1}\left|\begin{array}{cc} s_{p}\left(\lambda_{1}\right) & s_{q}\left(\lambda_{1}\right)\\ s_{p}^{'}\left(\lambda_{2}\right) & s_{q}^{'}\left(\lambda_{2}\right) \end{array}\right|.\; \end{array}$$

These formulae also apply when there are only three transition wavelengths. In this case let λ_4_ equal λ_max _.

When λ_1_ and λ_2_ are the only transition wavelengths, we have

$$\begin{array}{c} J_{\Phi }\left(\bar{\lambda }\right)=\left\{\begin{array}{cc} -s_{1}\left(\lambda_{1}\right) & s_{1}\left(\lambda_{2}\right)\\ -s_{2}\left(\lambda_{1}\right) & s_{2}\left(\lambda_{2}\right)\\ -s_{3}\left(\lambda_{1}\right) & s_{3}\left(\lambda_{2}\right) \end{array}\right\}, \end{array}$$

thus evaluation of the Jacobian matrix (46) becomes simpler.

To summarize, we start solving by i) setting a desired accuracy criterion; and ii) choosing some initial value $\bar{\lambda }{}^{0}$. As to the accuracy, we wish to find a value $\bar{\lambda }$, such that $d\left(\bar{\lambda }{}^{*},\bar{\lambda }\right)<ɛ$, where *d* is some metric, and ε is the criterion value. In particular, we used $d\left(\bar{\lambda }{}^{*},\bar{\lambda }\right)=\max\left\{\left|\lambda_{1}^{*}-\lambda_{1}\right|,\left|\lambda_{2}^{*}-\lambda_{2}\right|\right\}$. As to the initial value, we produced a set of initial values by computing the color signals $Z=\left\{\Phi \left(\lambda_{1}^{i},\lambda_{2}^{j}\right)\right\}$ for an even grid $\Lambda_{N}=\left\{\left(\lambda_{1}^{i},\lambda_{2}^{j}\right)\right\}$, *i*, *j* = 1,…*N*. Then, we computed the set of directions $A=\left\{\left(\alpha_{1}^{i},\alpha_{2}^{j}\right)\right\}$ corresponding to *Z*. When solving Equation 41, we take as the first initial value the one which corresponds to the direction in A closest to ${\bar{\alpha }}^{*}=\left(\alpha_{1}^{*},\alpha_{2}^{*}\right)$ corresponding to ${\bar{z}}^{*}$. Formally, the algorithm is as follows.

Step 1. Find the direction ${\bar{\alpha }}^{0}=\left(\alpha_{1}^{0},\alpha_{2}^{0}\right)$ in *A* closest (in terms of angular proximity) to ${\bar{\alpha }}^{*}$. Let $\bar{\lambda }{}^{0}=\left(\lambda_{1}^{0},\lambda_{2}^{0}\right)$ and, perhaps, λ_3_ and λ_4_ be the transition wavelengths of the optimal stimulus for direction ${\bar{\alpha }}^{0}$.

Step 2. Evaluate ${\bar{\lambda }}^{1}$ via Equation 44.

Step 3. Repeat step 2 using ${\bar{\lambda }}^{1}$ as the initial value. Specifically, given ${\bar{\lambda }}^{1}$, evaluate i) all the transition wavelengths (i.e., find λ_3_ and λ_4_, if any); and ii) ${\bar{\alpha }}^{1}=F\left(\bar{\lambda }{}^{1}\right)$. And then evaluate ${\bar{\lambda }}^{2}$ via Equation 45; and so on.

Stop when the difference between ${\bar{\lambda }}^{n}$ and ${\bar{\lambda }}^{n-1}$ is less than the desired accuracy criteria ε.
